# Priority recommendations for the implementation of patient-reported outcomes in clinical cancer care: a Delphi study

**DOI:** 10.1007/s11764-021-01135-2

**Published:** 2022-02-02

**Authors:** C. Mazariego, M. Jefford, R. J. Chan, N. Roberts, L. Millar, A. Anazodo, S. Hayes, B. Brown, C. Saunders, K. Webber, J. Vardy, A. Girgis, B. Koczwara, on behalf of the COSA PRO Working Group

**Affiliations:** 1 The Daffodil Centre, The University of Sydney, a joint venture with Cancer Council NSW, 153 Dowling street, Woolloomooloo, NSW 2011 Australia; 2 Department of Health Services Research, Peter MacCallum Cancer Centre, Melbourne, Victoria Australia; 3 Sir Peter MacCallum Department of Oncology, University of Melbourne, Melbourne, Victoria Australia; 4 Caring Futures Institute, College of Nursing and Health Sciences, Flinders University, Adelaide, South Australia Australia; 5 Metro North Health Service, Herston, QLD Australia; 6 University of Queensland Centre for Clinical Research, Herston, QLD Australia; 7 Medical School, University of Western Australia, Perth, WA Australia; 8 School of Women’s and Children’s Health, University of New South Wales, Randwick, Sydney, Australia; 9 Kids Cancer Centre, Sydney, Sydney Children’s Hospital, Randwick, Sydney, Australia; 10 Nelune Comprehensive Cancer Centre, Prince of Wales Hospital, Sydney, Australia; 11 Consumer representative, Patients First: The Continuous Improvement in Care-Cancer Project, Perth, Australia; 12 Wellbeing and Preventable Chronic Diseases Division, Menzies School of Health Research, Charles Darwin University, Brisbane, Australia; 13 School of Medical Sciences, Monash University, Clayton, Vic Australia; 14 Oncology Department, Monash Health, Clayton, Vic Australia; 15 Sydney Medical School, University of Sydney, Camperdown, Australia; 16 Concord Cancer Centre, Concord Hospital, Concord, NSW Australia; 17 Ingham Institute for Applied Medical Research, South Western Sydney Clinical School, University of New South Wales, Liverpool, New South Wales Australia; 18 Department of Clinical Oncology, Flinders Medical Centre, Adelaide, SA Australia; 19 Flinders Health and Medical Research Institute, Flinders University, Adelaide, SA Australia

**Keywords:** Patient-reported outcomes, PRO, Implementation, Quality of life, Survivorship, Health services

## Abstract

**Purpose:**

The aim of this study was to develop priority recommendations for the service level implementation of patient-reported outcomes (PROs) into clinical cancer care.

**Methods:**

Development of draft guidance statements was informed by a literature review, the Knowledge to Action (KTA) implementation framework, and discussion with PRO experts and cancer survivors. A two-round modified Delphi survey with key stakeholders including cancer survivors, clinical and research experts, and Information Technology specialists was undertaken. Round 1 rated the importance of the statements and round 2 ranked statements in order of priority.

**Results:**

Round 1 was completed by 70 participants with round 2 completed by 45 participants. Forty-seven statements were rated in round 2. In round 1, the highest agreement items (>90% agreement) included those that focused on the formation of strong stakeholder partnerships, ensuring ongoing communication within these partnerships, and the use of PROs for improvement and guidance in clinical care. Items ranked as the highest priorities in round 2 included assessment of current staff capabilities and service requirements, mapping of workflows and processes to enable collection, and using collected PROs to guide improved health outcomes.

**Conclusions:**

This stakeholder consultation process has identified key priorities in PRO implementation into clinical cancer care that include clinical relevance, stakeholder engagement, communication, and integration within the existing processes and capabilities.

**Implication for Cancer Survivors:**

Routine adoption of PRO collection by clinical cancer services requires multiple implementation steps; of highest priority is strong engagement and communication with key stakeholders including cancer survivors.

**Supplementary Information:**

The online version contains supplementary material available at 10.1007/s11764-021-01135-2.

## Introduction

The direct input from cancer patients and survivors on their needs, symptoms, and experiences of care is fundamental to the delivery of high quality, personalised cancer care [1, 2]. A standardised way to obtain such input is through the collection of patient-reported outcomes (PROs) that provide a ‘status report’ derived directly from patients [3]. PROs are typically collected using patient reported outcome measures (PROMs), which are questionnaires, ideally validated, across a range of health conditions and settings and used to assess overall general health, quality of life, psychological, emotional, and physical well-being [4, 5]. Collection of PROs has been extensively utilised in research, health service performance monitoring, and more recently, in direct cancer care.

### Collection of PROs

Benefits of PRO collection in clinical cancer care have been well established and include greater patient satisfaction with care [6], reduced emergency presentations [7, 8] and improved survival [7, 9]. The use of PROs has also been shown to support patient-centred care through increased patient activation [3], predict treatment outcomes and complications [10, 11], and assist in monitoring of the quality and safety with health services [12].

Despite these many benefits, the use of routine PROs in clinical cancer care remains limited [13, 14].To facilitate adoption, health services need explicit recommendations regarding effective implementation of PRO collection systems.

The International Society for Quality of Life Research (ISOQOL) recommended minimum standards for the implementation of PROs used in patient-centred outcomes [15–17]. These recommendations stated that PRO initiatives were more likely to be successful if integrated into clinical workflow through direct engagement with all stakeholders, including patients, who would be involved in PRO collection [15–17]. A case-series paper further discussed the importance of using implementation science through a multi-level approach [17]. It found that while identified barriers to the implementation of routine collection of PROs were consistent across different health settings, including cancer, enablers were context specific and further work was necessary to develop practical steps for the design of implementation strategies.

While this case-series paper provided a starting point from which to consider PRO implementation more broadly, there remains a need for practical recommendations that use implementation science theory and the real-world perspective of stakeholders. To address this need, the present study aimed to develop priority recommendations for the implementation of PROs into routine clinical cancer care at the health service level. This work first considered the level of importance of implementation factors and then their relative priority resulting in recommendations for implementation that a cancer service should consider when attempting to implement the collection of PROs.

## Methods

The study employed a modified Delphi methodology—involving a series of iterative questionnaires to determine relative priorities for implementation [18–20]. The Delphi process included four characteristic features: anonymity, iteration with controlled feedback, statistical group response, and expert input [18–20].

### Participant recruitment

Participant recruitment included those potentially engaged with PRO implementation (e.g. healthcare professionals [patient facing roles in which cancer care is provided], information technology (IT) staff, hospital administrators, and researchers, as well as cancer survivors). A multidisciplinary working group (WG) of experts in PROs, and cancer patients and survivors, had been established by the Clinical Oncology Society of Australia (COSA) to advocate for and promote the use of PROs in cancer, and was invited to provide input as to the design of the study. Following a COSA think tank on PRO use in Australia in 2018, a working group of interested clinicians, researchers, and consumers was formed through an open invitation to members and other interested individuals, to further advance the PRO implementation agenda in Australia. The group published a ‘call to action’ in the Medical Journal of Australia, which advocated for the advancement of personalised cancer care through the collection of PROs [1]. The need for the Delphi study on implementation was identified as the next step. For this Delphi study, participants were recruited by distributing our survey through the clinical and consumer networks of the WG. WG members were sent an invitation email (including the participant information sheet and the link to the study consent form) to complete the Delphi survey, and this was further distributed across their networks using a snowball approach. This WG has wide representation of key stakeholders in cancer survivorship clinical care as members are representatives of a variety of care cancer units/organisations (e.g. tertiary care centres, community care centres) and patient advocate groups across Australia.

Within the survey link, participants were asked to provide consent, basic demographic information, and a contact email. These contact emails were used for round 2 survey distribution, as only participants who engaged in round 1 were invited to participate in round 2. No incentives were provided to facilitate further engagement between rounds. However, two reminder emails were sent to participants of round 1 to complete round 2.

### Generation of priority statements

The development of statements used in the Delphi process was informed by a literature review examining implementation of PROs in cancer care settings, the Knowledge to Action (KTA) implementation framework [21], and feedback from the WG.

The study team reviewed literature through keyword searches in PubMed and Medline for articles addressing the implementation of PROs in cancer care settings (Appendix A). The drafting of priority statements occurred iteratively with the literature review. Articles were identified as being relevant if they included either considerations of barriers and/or enablers for the implementation of PROs or discussed points of consideration when deciding how best to implement PROs into clinical practice (e.g. position statements on PRO implementation). Statements were first drafted as close to verbatim from the derived literature. Next, the KTA was utilised as a framework through which to categorise emerging priorities into domains. The KTA is an implementation process model that describes practical steps or stages in translating research into practice, with core concepts of knowledge and creation [21]. The framework outlines six domains for implementation consideration: (1) adapt knowledge to local context, (2) address barriers, (3) develop implementation strategies, (4) monitor use context/adaptations & designing tests of change, (5) evaluate outcomes, and (6) focus on sustainability.

To consolidate the list of statements prior to the first round of the Delphi study, items under each of the KTA domains were reviewed, refined, discussed, and examined for clarity and understanding by the WG.

### Priority review process

Drafted statements underwent a two-round modified Delphi process. The online consensus process was facilitated through a survey-based platform (RedCap V10.0.1).

An invitation email (Appendix B) including the participant information sheet (Appendix C) and link to the round 1 Delphi survey was sent to the WG for dissemination. This was distributed by the WG using a nomination and snowball sampling approach. Only participants who engaged in round 1 were invited to participate in round 2.

### Round 1—rating of importance

In round 1, participants were asked to rate the importance of each item for the implementation of routine collection of PROs at the health service level using a 9-point Grading of Recommendations Assessment, Development and Evaluation (GRADE) scale [22]. Scores of 7–9 indicated the item was of ‘critical importance’, 4–6 indicated ‘importance’, and 1–3 indicated ‘limited importance’. Free text fields were provided for each survey section encouraging participants to comment on or explain their ratings, and to add or rate new items that they felt were not already covered. The round 1 survey is available as Appendix D. At the conclusion of round 1, the WG was convened to discuss the results and to provide feedback on which items should progress to ranking in round 2.

### Round 2—priority ranking

In the second round, participants were presented with the results of round 1 and asked to rank the presented statements in order of importance with ‘1’ being the highest priority item to consider when attempting to implement PROs at the health service level. Free-text fields were provided to suggest additions and/or comments. Round 2 survey is available as Appendix E.

### Priority review meeting

The WG convened a final priority review meeting to discuss study results and the associated priority ratings of each item and to discuss generalisability and implications for policy and future research.

### Statistical analysis

The study team synthesised the agreement levels of each statement from round 1 through descriptive statistics of agreement levels per item. An additional, conventional content analysis of the free-text fields was undertaken to capture recommended modifications or adjustments to presented statements [23]. Statements that received a ranking of 7–9 from ≥70% of participants and a ranking of 1–3 by ≤ 15% of participants were deemed to have high priority levels [22]. Otherwise, the statement was considered to need re-drafting using either qualitative feedback obtained or WG input. Summary reports of all items included in the Delphi round 1 were produced and disseminated at the start of round 2 to all survey participants for reference and consideration.

Upon completion of Delphi round 2, median ranking scores and interquartile ranges were calculated for each statement. Further assessment of free-text field comments through content analysis was completed and presented where relevant to complement the interpretation of the quantitative statistical results.

This study was approved by the Cancer Council NSW Ethics Committee (ref. #324).

## Results

### Priority statement generation

Our targeted review search identified one position statement on how to best integrate PROs into clinical practice [24], several scoping/systematic/pooled reviews that discussed implementation of PROs [6, 25–31], and five methodological and research articles that discussed using implementation approaches to implement and evaluate PRO use in clinical practice [5, 9, 32–34]. These sources led to the development of 71 suggested statements (Appendix F).

Statements were organised conceptually into five domains of the KTA framework, combining domains four and five ( [4] monitor use context/adaptations & designing tests of Change and [5] evaluate outcomes) as few statements arose in these domains and those that did were deemed to be complementary to each other. Through WG input and consolidation, 47 statements were next formatted into questionnaire items that formed the first round of the Delphi study.

### Priority review process

A total of 70 PRO stakeholders participated in round 1, with 45 continuing to round 2. Table 1 summarises the characteristics of participants. The majority of participants in round 1 were healthcare professionals (51.4%, *n*=36/70), predominantly medical oncologists (30.8%), treating adult patients (41.0%). Round 2 encapsulated a similar distribution of participants.

**Table 1:** Characteristics of Delphi participants across both rounds

	Round 1		Round 2	
Demographic variable/question	*N*	%	*N*	%
	*N*=70	100	*N*=45	100
Primary role				
Healthcare professional	36.0	51.4	20.0	44.4
Academic/Scientific staff (e.g. laboratory scientist)	3.0	4.3	3.0	6.7
Health service administrator (e.g. health service manager, CEO)	9.0	12.9	6.0	13.3
Researcher involved with PRO implementation	14.0	20.0	11.0	24.4
IT professional/health IT developer	3.0	4.3	1.0	2.2
Consumer- person with a personal experience with cancer, a carer for person with cancer, a family member of someone with cancer	5.0	7.1	4.0	8.9
If healthcare professional, population of patient focus*				
Haematology	12.0	15.4	7.0	15.9
Medical oncology	24.0	30.8	15.0	34.1
Radiation oncology	17.0	21.8	7.0	15.9
Cancer surgery	5.0	6.4	3.0	6.8
Bone marrow transplant	4.0	5.1	2.0	4.6
If healthcare professional, age group of patient focus*				
Paediatric	4.0	5.1	2.0	4.6
Adolescents/young adults	9.0	11.5	4.0	9.1
Adults	32.0	41.0	17.0	38.6
Geriatric	8.0	10.3	4.0	9.1
Familiarity with PRO collection				
Expert (very familiar with PROs and regularly uses or supports use of PROs)	29.0	41.4	23.0	51.1
Intermediate (some familiarity with PROs, moderate usage/support)	26.0	37.1	12.0	26.7
Novice (new to PRO use)	15.0	21.4	10.0	22.2
If not novice, role in PRO collection/use*				
System builder (building infrastructure to collect PROs)	23.0	29.5	15.0	34.1
Collector or analyst of PROs	37.0	47.4	24.0	54.6
Participant providing input	6.0	7.7	4.0	9.1
State/territory				
NSW	22.0	31.4	16.0	35.6
SA	1.0	1.4	1.0	2.2
VIC	4.0	5.7	4.0	8.9
QLD	21.0	30.0	9.0	20.0
WA	22.0	31.4	15.0	33.3
Experience (delivering or receiving) cancer care in rural and remote areas				
No	42.0	60.0	29.0	65.9
Yes	28.0	40.0	15.0	34.1
Primary setting at which care is delivered or received				
Public hospital	55.0	78.6	31.0	68.9
Private hospital	1.0	1.4	1.0	2.2
Private specialist practice	2.0	2.9	2.0	4.4
Other	12.0	17.1	11.0	24.4

*Multiple answers were permitted

Abbreviations: *CEO*, Chief Executive Officer; *PRO*, patient reported outcome; *IT*, information technology; *NSW*, New South Wales; *SA*, South Australia; *VIC*, Victoria; *QLD*, Queensland; *WA*, Western Australia

The majority of participants identified their familiarity with PROs as either ‘expert’ (41.4%, *n*=29/70) or ‘intermediate’ (37.1%, *n*=26/70) users. Of these, the majority labelled themselves as PRO collectors or analysts (47.4%), followed by PRO collection ‘system builders’ or involved in the design of infrastructure to routinely collect PROs (29.5%), and participants in PRO collection (7.7%).

Participants were from five of eight Australian states and territories with the majority residing in New South Wales and Western Australia (31.4%, *n*=22/70 each).

### Round 1—rating of importance

After round 1, seven statements did not meet the agreed threshold for ‘high priority’ rating (range 62–69%, statements denoted with an asterisk in Table 2). However, as the process of the modified Delphi was not to exclude items that had not met general consensus, but rather to identify the emerging priority rankings of the statements, all statements were retained for round 2. Participants provided a total of 29 free-text field comments, the majority of which (*n*=22) were general statements of agreement with the presented items or highlighted the item’s importance. A few comments (*n*=7) suggested reasons for disagreement with items relating to clinician prompting for PRO collection. No new items were recommended nor modifications to existing items suggested.

**Table 2:** Results from round 1 (importance) and round 2 (ranking) across both Delphi rounds by domain of Knowledge to Action framework

	**Round 1—importance**			**Round 2—ranking**	
Consensus statements	Not important	Important	Critical	Median rank^+^	Interquartile range (Q1–Q3)
	(%)	(%)	(%)		
**Section 1: Readiness to implement PROs**					
1.1 Conduct a formal assessment of the willingness of the organisation to implement PROs	0	20.9	77.6	5.0	(3.0–11.0)
1.2 Understand current beliefs and attitudes of staff related to PRO use	0	22.4	11.6	6.0	(3.0–10.0)
1.3 Identify current staff capabilities, skills and service requirements to implement routine PRO collection	0	10.5	89.6	4.5	(2.0–7.0)
1.4 Assess existing resources and infrastructure that can be deployed to aid the collection of PROs	0	9	91	5.0	(3.0–7.3)
1.5 Conduct an assessment of patient and clinician willingness to participate in PRO collection	3	25.4	71.7	6.0	(3.0–10.0)
1.6 Determine which populations of patients should be invited to participate in PRO collection*	4.5	26.9	68.7	10.0	(7.0–11.3)
1.7 Ensure equity, diversity and inclusion when determining which patients will be invited to participate in PRO collection	1.5	22.4	76.1	9.0	(7.0–11.0)
1.8 Identify potential local barriers and facilitators that will help or hinder PRO implementation	0	10.5	89.6	5.0	(3.8–7.3)
1.9 Form strong partnerships with stakeholders who will be involved in PRO collection and use (e.g. consumers, clinicians, IT/infrastructure developers, health service administrators)	0	7.5	92.5	5.0	(2.0–7.3)
1.10 Consult with relevant stakeholders that will be impacted by the routine implementation of PROs (i.e., patients, family members, clinicians, administration, IT, service administrators) to discuss strategies for implementation	0	17.9	82.1	6.0	(2.8–8.0)
1.11 Establish implementation teams that incorporate all stakeholders (mentioned above) to guide the implementation of PROs	0	13.4	86.6	6.5	(3.0–7.3)
1.12 Assess, with stakeholder consultation, when PROs should be collected	0	11.9	88.1	11.0	(8.0–12.0)
1.13 Consider if additional resourcing streams are necessary to enable the implementation of PRO collection	0	10.5	88.1	10.0	(7.8–13.0)
**Section 2: Addressing barriers**					
2.1 Clearly articulate the evidence for and value of using PROs to all stakeholders	1.5	9.1	89.4	3.0	(1.0–6.0)
2.2 Select which existing validated tools (surveys) should be used for the collection of PROs	0	18.2	81.8	3.5	(2.0–7.3)
2.3 Decide on the appropriate format for PRO collection (i.e. paper based or electronic)	0	27.3	72.7	4.0	(3.0–8.3)
2.4 Establish how PRO data will be stored and who will have access to the data	0	19.7	80.3	7.0	(4.0–11.0)
2.5 Map workflows and processes needed for PRO implementation	0	10.6	89.4	2.5	(1.0–4.3)
2.6 Develop systems that will allow the PRO information to be used for quality improvement	0	16.7	83.3	8.0	(5.0–11.3)
2.7 Develop systems that will allow the PRO information to be used for performance measurement*	4.6	31.8	62.1	12.0	(6.8–15.0)
2.8 Develop systems that will allow the PRO information to be used for research*	1.5	28.8	69.7	11.0	(8.0–13.3)
2.9 Provide training for clerical staff on the role of PROs and local administrative processes associated with their collection	3	18.2	78.8	9.0	(6.0–12.0)
2.10 Run dedicated training sessions for clinicians on how to utilise and interpret PRO reports	0	22.7	77.3	8.0	(6.0–10.0)
2.11 Run education programs to help patients and families who are first entering the cancer system understanding why PROs are being collected and how to use the PRO platform/system (as appropriate)*	6.1	31.8	62.1	10.5	(7.0–13.0)
2.12 Ensure patient-centred education programs (item above) will include why PROs are collected and how PROs can improve their health outcomes*	6.1	25.8	68.2	11.0	(7.5–13.0)
2.13 Develop guidelines to help clinicians respond to patient concerns and issues identified in collected PRO data	0	10.6	81.8	7.0	(4.8–10.0)
2.14 Supply patients and families with guidance about actions to take regarding PRO results	3	18.2	78.8	10.0	(7.0–13.0)
2.15 Develop and adopt clinician-facing and patient friendly reports that summarise PRO scores for ease of interpretation and actionability	1.5	13.7	84.9	8.0	(3.8–10.0)
**Section 3: Developing implementation strategies**					
3.1 Decide on an implementation approach using an implementation framework*	1.5	27.3	69.7	4.0	(2.0–6.3)
3.2 Identify and support champions within the organisation who can drive the implementation of PROs	0	13.6	86.4	2.0	(2.0–4.0)
3.3 Seek feedback from patients regarding the process of PRO collection and use of data in clinical care, and use this feedback to guide further initiatives	0	25.8	74.2	6.0	(4.8–7.0)
3.4 Integrate PROs into clinical workflow, care pathways, and the way team members work together so that the use of PROs becomes routine practice	0	10.6	89.4	2.0	(1.0–3.3)
3.5 Establish a centralised system to deliver technical assistance focused on the implementation of PROs	1.5	21.2	75.8	6.0	(4.0–7.0)
3.6 Pilot the collection of PROs in a smaller controlled group of patients before service wide rollout	6.1	15.2	77.3	4.0	(2.8–5.0)
3.7 Develop reminder systems to help prompt clinicians to collect PROs*	7.6	22.7	68.2	7.0	(5.0–8.0)
3.8 Identify and learn from other sites where the collection of PROs has been successful	0	13.6	86.4	4.0	(3.0–6.0)
**Section 4: Monitoring use and evaluating outcomes**					
4.1 Collect and summarise clinical performance data and report performance back to clinicians and administrators	1.5	21.2	77.3	3.5	(2.8–5.0)
4.2 Monitor how many clinicians/clinics are collecting and using PROs	1.5	27.3	71.2	4.0	(2.0–5.0)
4.3 Evaluate the reasons for non-use of PROs in clinical care	0	16.7	83.3	4.0	(3.0–6.0)
4.4 Record any changes to clinical practice once PROs are implemented	0	7.6	92.4	3.5	(2.0–5.0)
4.5 Ensure ongoing communication with administrative, clinical, Information Technology staff, and patients to understand their evolving needs in collecting PROs	0	7.6	92.4	4.0	(2.0–5.0)
4.6 Monitor patient and clinician engagement with PROs once they are implemented for quality assurance and intervention refinement	1.5	15.2	83.3	2.0	(1.0–3.0)
**Section 5: Sustainability**					
5.1 Conduct an assessment of what is necessary for long-term continuation of routine PRO collection	0	10.6	89.4	3.0	(1.0–5.0)
5.2 If appropriate, use collected PROs to guide and improve clinical care at the health service level	1.5	6.1	92.4	2.0	(1.0–4.0)
5.3 Continue regular training of health service staff in PRO collection and response	0	22.7	77.3	3.0	(2.8–5.0)
5.4 Identify dedicated resources that are needed to keep PRO collection ongoing	0	12.1	87.9	2.0	(2.0–4.0)
5.5 Develop a protocol for PRO collection that is regularly evaluated and refined when necessary	0	21.2	78.8	3.0	(3.0–4.0)

*Items that did not meet 70% agreement in round 1, ^+^ lower scores= higher levels of importance. Grading of Recommendations Assessment, Development and Evaluation (GRADE) scales^16^ were used for round 1 results, scaling from ‘1’ to ‘9’. Scores of 7–9 indicated the item was of ‘critical importance’, scores of 4–6 indicated ‘importance’, and scores of 1–3 indicated ‘limited importance’. Abbreviations: *PRO*, patient reported outcome; *IT*, information technology

### Round 2—priority ranking

Round 2 rankings revealed the relative importance of statements; the top five statements for each section of the KTA are summarised in Table 3.

**Table 3:** Top 5 most highly prioritised statements within each Knowledge To Action domain to consider when attempting to implement PROs into routine clinical cancer care practice

**Section 1: Readiness to implement PROs**	
1. Identify current staff capabilities, skills and service requirements to implement routine PRO collection	
2. Form strong partnerships with stakeholders who will be involved in PRO collection and use (e.g. consumers, clinicians, IT/Infrastructure developers, health service administrators)	
3. Assess existing resources and infrastructure that can be deployed to aid the collection of PROs	
4. Consult with relevant stakeholders that will be impacted by the routine implementation of PROs (i.e., patients, family members, clinicians, administration, IT, service administrators) to discuss strategies for implementation	
5. identify potential local barriers and facilitators that will help or hinder PRO implementation	
**Section 2: Addressing barriers**	
1. Map workflows and processes needed for PRO implementation	
2. Clearly articulate the evidence for and value of using PROs to all stakeholders	
3. Select which existing validated tools (surveys) should be used for the collection of PROs	
4. Decide on the appropriate format for PRO collection (i.e. paper based or electronic)	
5. Develop guidelines to help clinicians respond to patient concerns and issues identified in collected PRO data	
**Section 3: Developing implementation strategies**	
1. Identify and support champions within the organisation who can drive the implementation of PROs	
2. Integrate PROs into clinical workflow, care pathways, and the way team members work together so that the use of PROs becomes routine practice	
3. Pilot the collection of PROs in a smaller controlled group of patients before service wide rollout	
4. Identify and learn from other sites where the collection of PROs has been successful	
5. Decide on an implementation approach using an implementation framework	
**Sections 4 & 5: Monitoring use and evaluating outcomes**	
1. Monitor patient and clinician engagement with PROs once they are implemented for quality assurance and intervention refinement	
2. Ensure ongoing communication with administrative, clinical, Information Technology staff, and patients to understand their evolving needs in collecting PROs	
3. Record any changes to clinical practice once PROs are implemented	
4. Collect and summarise clinical performance data and report performance back to clinicians and administrators	
5. Monitor how many clinicians/clinics are collecting and using PROs	
**Section 6: Sustainability**	
1. If appropriate, use collected PROs to guide and improve clinical care at the health service level	
2. Identify dedicated resources that are needed to keep PRO collection ongoing	
3. Conduct an assessment of what is necessary for long-term continuation of routine PRO collection	
4. Develop a protocol for PRO collection that is regularly evaluated and refined when necessary	
5. Continue regular training of health service staff in PRO collection and response	

Abbreviations: *PRO*, patient reported outcome; *IT*, Information Technology

The majority of free-text comments described difficulties that participants had ranking the importance of the presented statements (*n*=10/15), as they considered them to be of similar importance. As such, these participants suggested that their ranking process was instead a reflection of preferred sequential order when implementing PROs at the health service level.

### Priority review meeting

The final priority review meeting, attended by 10 of the 15 members of the WG, resulted in unanimous agreement on the top five statements within each domain being of the highest priority for a health service to consider first when attempting to implement a process for routine collection of PROs (Table 3). The WG discussed intentions for future national advocacy for the incorporation of these findings into the national cancer plan.

## Discussion

This is the first study that reports on what multidisciplinary users, including cancer patients, health care providers, administrators, and IT professionals, consider as priority actions for implementation of PROs into clinical cancer care.

Based on this work, the key practical step for PRO implementation is for health services to identify current staff capabilities and the services required to undertake the collection of PROs. Next, health services should strive to form strong stakeholder partnerships with those who will be involved with PRO collection and use, especially cancer survivors. Our recommendations further discuss which barriers were seen as priorities to address, with mapping workflow and process and the need for clear articulation as to the evidence behind the value of PRO collection deemed as highest priority. The most important considerations in the development of implementation strategies were to identify support champions and to practically design the integration of PROs in care pathways and clinical workflows. Considered from the perspective of stakeholders, using PROs as quality assurance tools was deemed as an acceptable method of monitoring and evaluating outcomes of PRO implementation. Finally, using routinely collected PRO data to guide and improve clinical care at the health service level was viewed as the most critical priority to ensure sustainability of PRO collection.

The priorities identified in the present study are consistent with those identified by others, notably, the Canadian position statement [24]; however, the Canadian recommendations were derived from health professionals or academic researchers and were not ranked in terms of prioritisation of which items to consider first.

The present study extends the recommendations of the ISOQOL case-series reports [13, 17] by ranking the relevant implementation strategies necessary for PROs implementation from the perspective of the multidisciplinary stakeholders involved.

While previous literature on PRO implementation discussed the barriers and enablers of implementation [6, 15, 26–31], the present study provides a new lens through which to consider those barriers and enablers in terms of their relative importance to PRO stakeholders.

The majority of presented items were rated highly during round 1, and the prioritisation rankings in round 2 did not elicit much variation. This lack of outlying variation within our results may be due to our use of the KTA framework. Using this tool allowed for full consideration of all factors relating to implementation, but it may have led to naturally high ratings and low variation within ranking as the domains themselves are evidenced-based. As such, content within the domains would fundamentally be deemed important or of high priority from the outset.

Additionally, it is notable that most of our study’s recommendations focused on the initial set-up and adoption with less attention to sustainability. This may reflect the current state of the field and limited long-term use of PROs in clinical care. Literature suggests that sustainability in PRO collection would be best placed for success if the processes are designed through collaborative efforts with key stakeholders, especially cancer survivors [35, 36] and indeed our ranking, placed particular importance on stakeholder engagement and collaboration at the outset, although did not explicitly connect this to the sustainability outcomes.

### Strengths and limitations

Our study strengths lie within the representation of a variety of stakeholder perspectives including cancer survivors. We note however that our sample was overly representative of healthcare professionals, with a limited practical perspective from PRO system builders (e.g. IT specialists). While more specific data on the type of categorisation of healthcare professionals was not collected (e.g. medical doctor, nurse, physiotherapist, psychologist, etc.), our study objective of developing priority recommendations for the implementation of PROs into routine clinical cancer care at the health service level required a general healthcare professional perspective, irrespective of these sub-categorisations. Additionally, the attrition of participants from round 1 to round 2 may have impacted our round 2 results as participants were asked to rank statements in order of importance rather than their general agreement of importance (as they had in round 1). Higher participation numbers in round 2 may have produced a different ranking for statements depending on the personal attributes of the sample; however, this loss to follow-up in round 2 produced no major changes in terms of proportional representation amongst the categories of role groups.

Furthermore, this work was undertaken with the Australian context in mind and facilitated through Australian-based participants and thus may not be generalisable to all health settings or contexts with very different health care systems to the Australian model. While our results were contextually derived in the Australian setting, we believe they have international relevance as the KTA framework used to organise and consider implementation factors is applicable globally [21] and the similarity of our findings to other international recommendations attests to their global relevance.

Despite the limitations, this study stands as the first of its kind in formulating stakeholder-derived recommendations and a practical guide to implementing PROs in the cancer care clinical context. This work can be viewed as a building block from which health services can guide their considered efforts for PRO implementation while considering their local context.

## Conclusion

This stakeholder consultation process has identified key priorities in PRO implementation into clinical cancer care that include clinical relevance, stakeholder engagement, communication, and integration within the existing processes and capabilities. Future research should focus on pragmatic application of these recommendations to ensure growing uptake of PROs into clinical practice.

## Supplementary information

ESM 1 (DOCX 3054 kb)

## Data Availability

The datasets generated during and/or analysed during the current study are not publicly available due to conditions set out by the approving ethics committee but are available from the corresponding author on reasonable request.

## Code availability

N/A
