# Supplementary material for: Priority recommendations for the implementation of patient-reported outcomes in clinical cancer care: a Delphi study
Source: J Cancer Surviv. 2022 Feb 2;16(1):33–43. doi: 10.1007/s11764-021-01135-2 (PMC8881271; doi:10.1007/s11764-021-01135-2)
Supplement: Supplementary file 1 — (DOCX 3054 kb) [file 11764_2021_1135_MOESM1_ESM.docx]

**Priority Recommendations for the Implementation of Patient Reported Outcomes in Clinical Cancer Care: A Delphi study**

**SUPPLEMENTARY APPENDIX**

Mazariego C^1^, Jefford M^2,3^, Chan RJ^4^, Roberts N^5,6^, Millar L^7^, Anazodo A^8-10^, Hayes S^11^,  Brown B^12^, Saunders C^7^, Webber K^13,14^, Vardy J^15,16^, GirgisA^17^,  Koczwara B^18,19^, on behalf of the COSA PRO Working Group

1. The Daffodil Centre, The University of Sydney, a joint venture with Cancer Council New South Wales
2. Department of Health Services Research, Peter MacCallum Cancer Centre, Melbourne, Victoria, Australia
3. Sir Peter MacCallum Department of Oncology, University of Melbourne, Melbourne, Victoria, Australia
4. Caring Futures Institute, College of Nursing and Health Sciences, Flinders University, South Australia.
5. Metro North Health Service, Herston, QLD
6. University of Queensland Centre for Clinical Research, Herston, QLD
7. Medical School, University of Western Australia, Perth, WA, Australia
8. School of Women’s and Children’s Health, University of New South Wales, Randwick, Sydney, Australia
9. Kids Cancer Centre, Sydney, Sydney Children’s Hospital, Randwick, Sydney, Australia
10. Nelune Comprehensive Cancer Centre, Prince of Wales Hospital, Sydney, Australia
11. Consumer representative, Patients First: The Continuous Improvement in Care-Cancer Project
12. Wellbeing and Preventable Chronic Diseases Division, Menzies School of Health Research, Charles Darwin University
13. School of Medical Sciences, Monash University, Clayton, Vic, Australia
14. Oncology Department, Monash Health, Clayton, Vic, Australia
15. Sydney Medical School, University of Sydney, Australia
16. Concord Cancer Centre, Concord Hospital, NSW, Australia
17. Ingham Institute for Applied Medical Research, South Western Sydney Clinical School, University of New South Wales, Liverpool, New South Wales, Australia
18. Department of Clinical Oncology, Flinders Medical Centre, Adelaide, SA, Australia
19. Flinders Health and Medical Research Institute, Flinders University, Adelaide, SA, Australia

**Corresponding author:**

C G Mazariego

The Daffodil Centre

153 Dowling street, Woolloomooloo NSW 2011

02 9334 1366

[carolynma@nswcc.org.au](mailto:carolynma@nswcc.org.au)

Table of Contents

**Appendix A:** Keyword search strategy**3**

**Appendix B**: Delphi study Invitation email**4**

**Appendix C**: Delphi Participant Information Sheet **5**

**Appendix D:** Round 1 survey **7**

**Appendix E:** Round 2 survey.**19**

**Appendix F:** Draft priority statement generation list from literature review on PRO implementation.**30**

**Appendix A:** Keyword search strategy


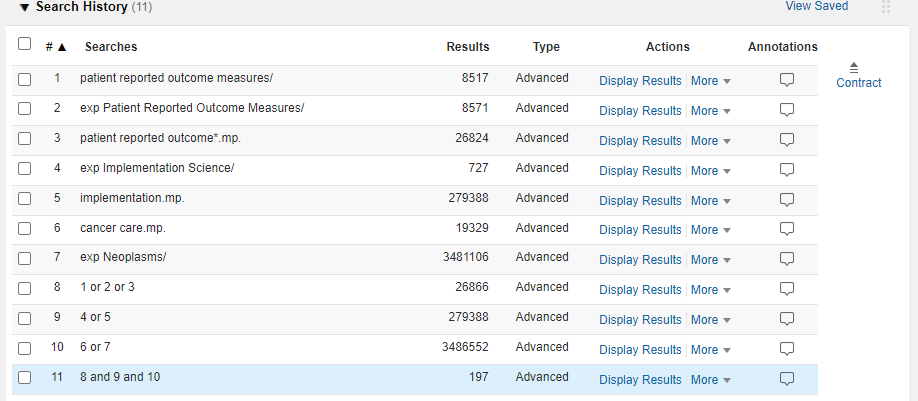


**Appendix B**: Delphi study Invitation email


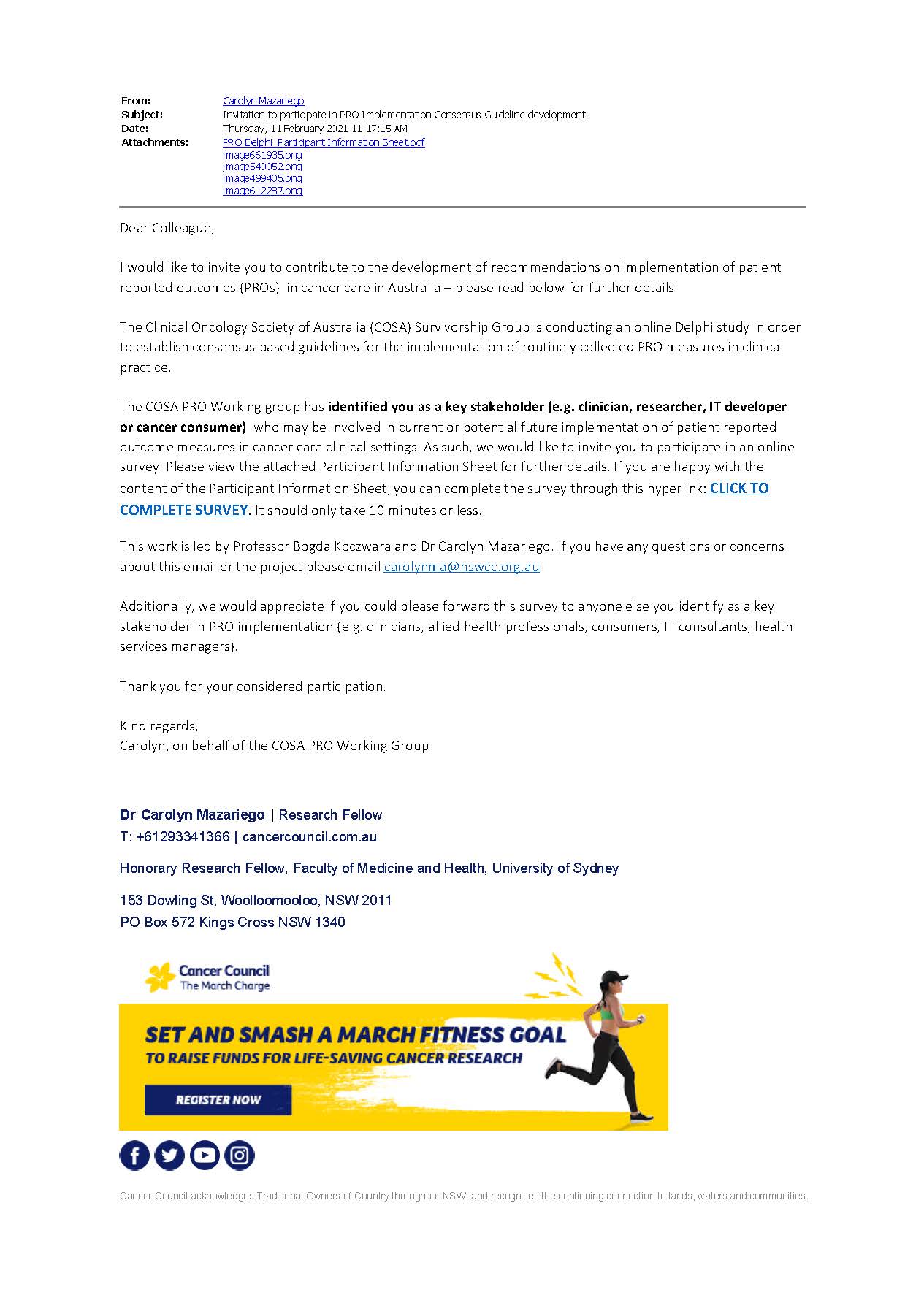


**Appendix C**: Delphi Participant Information Sheet
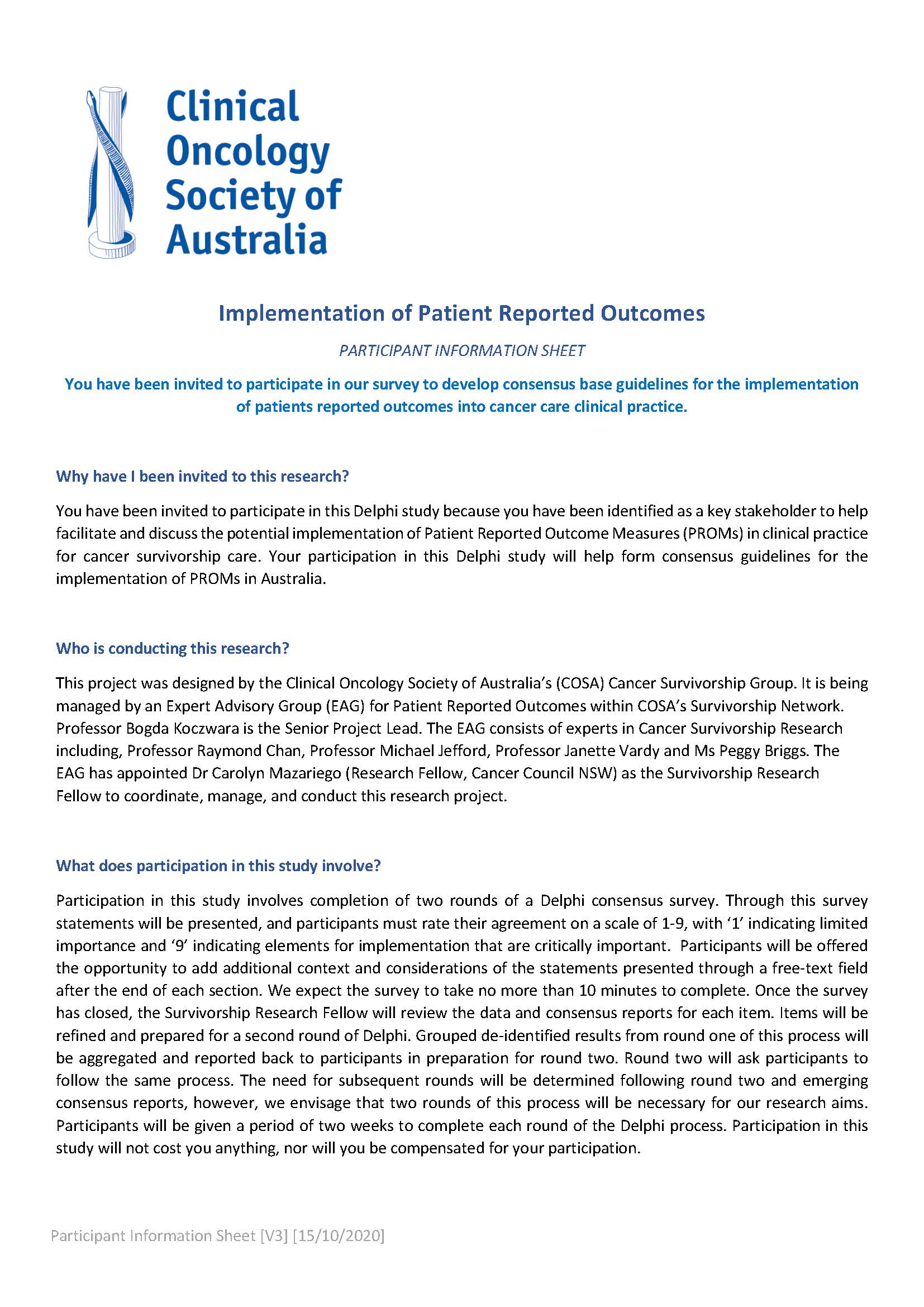

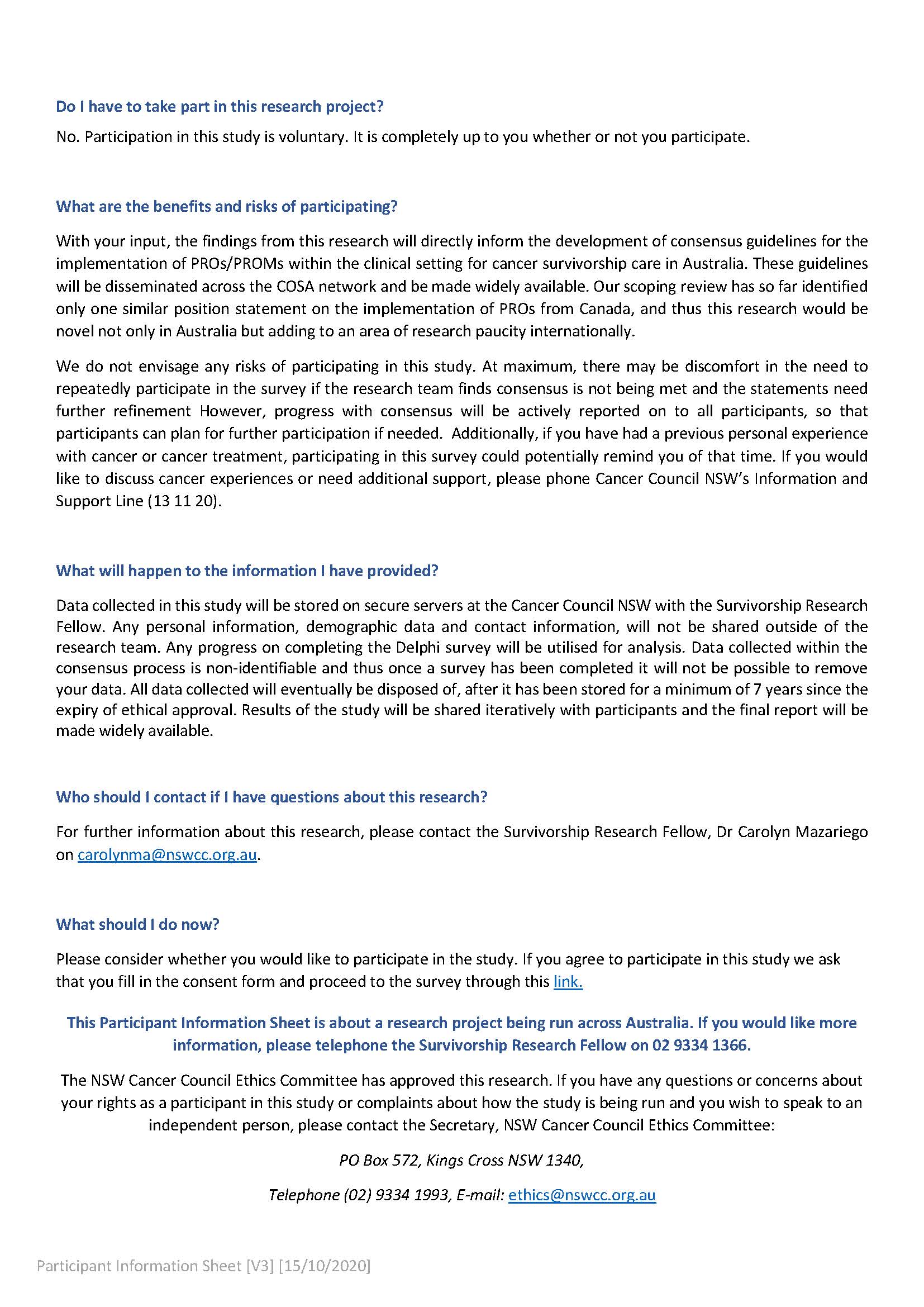


**Appendix D:** Round 1 survey
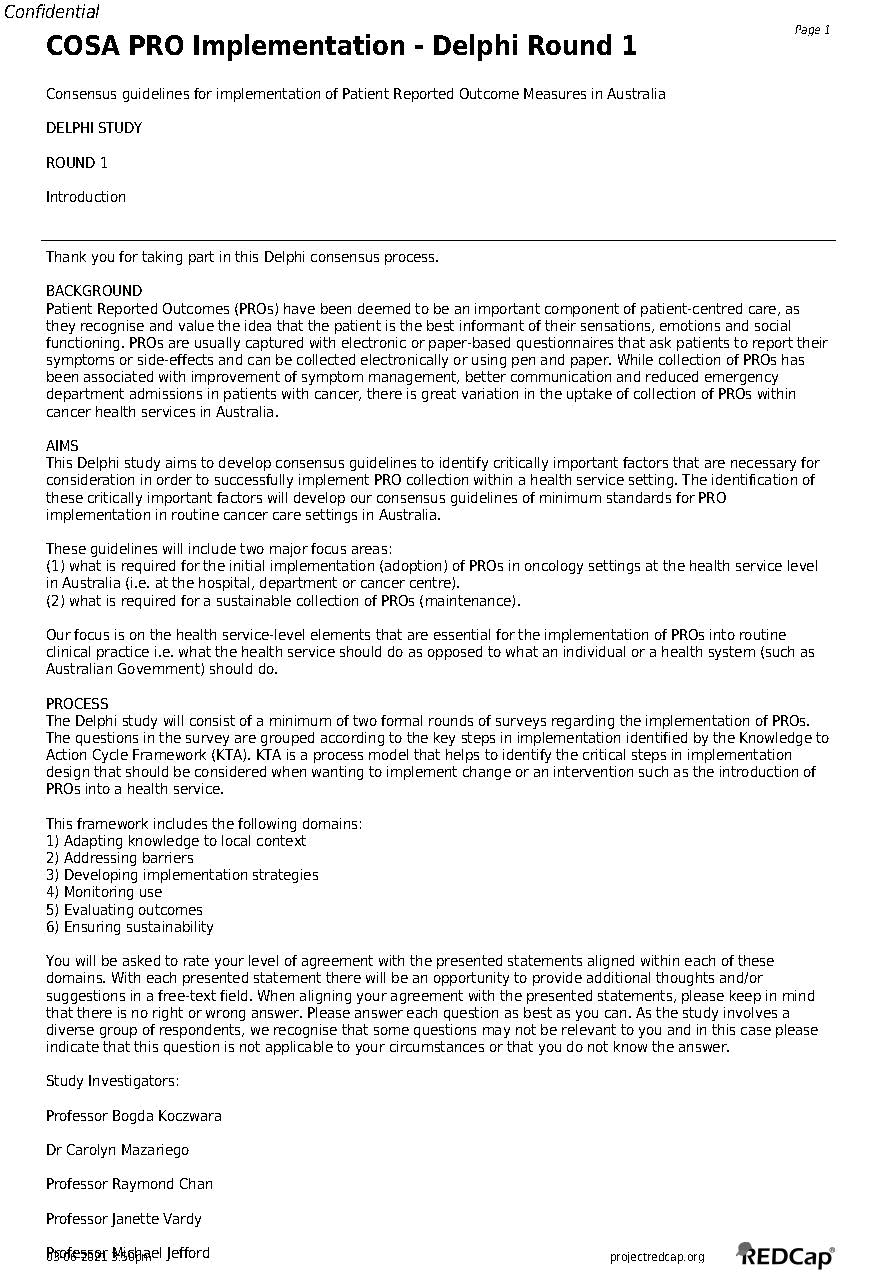

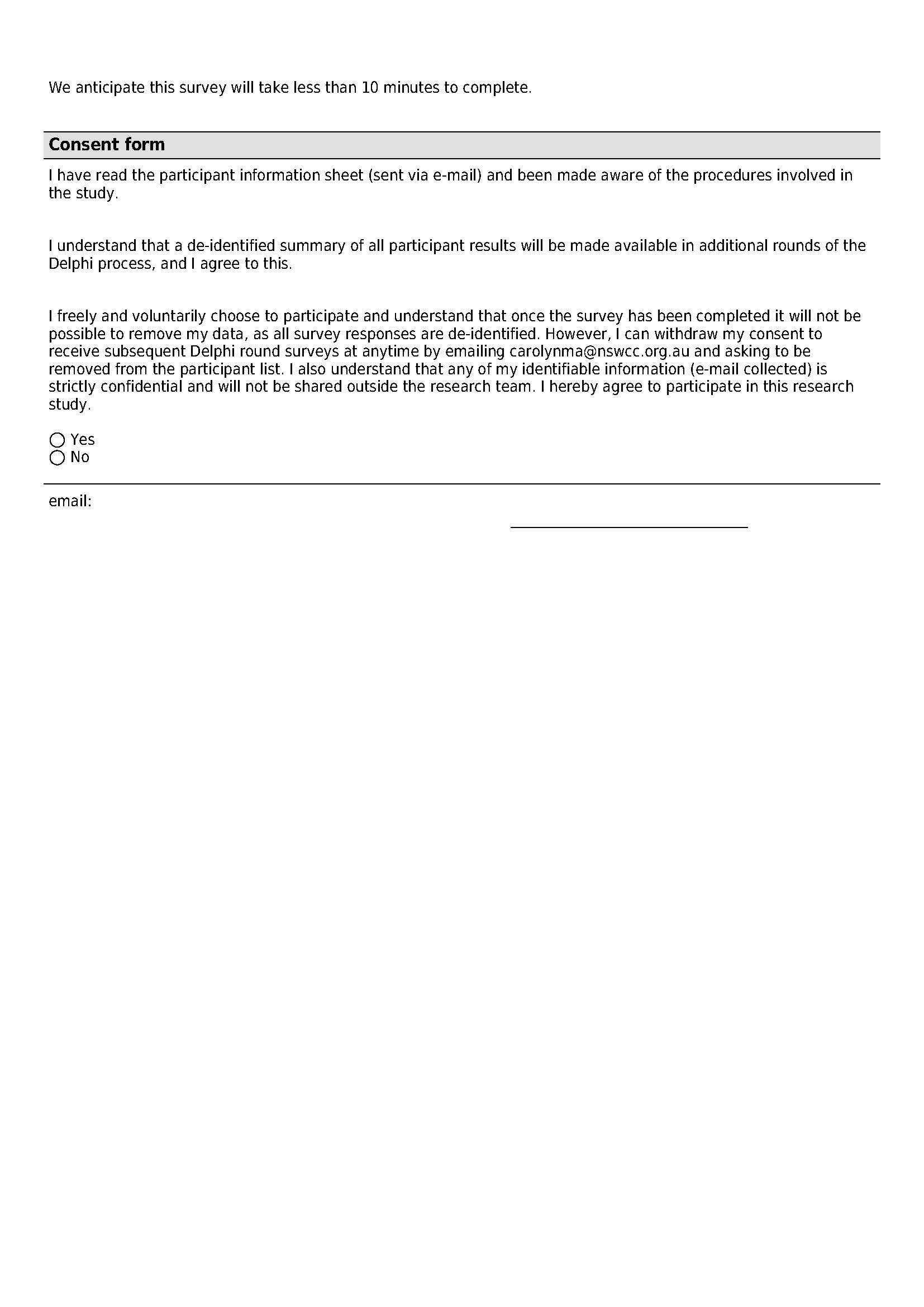

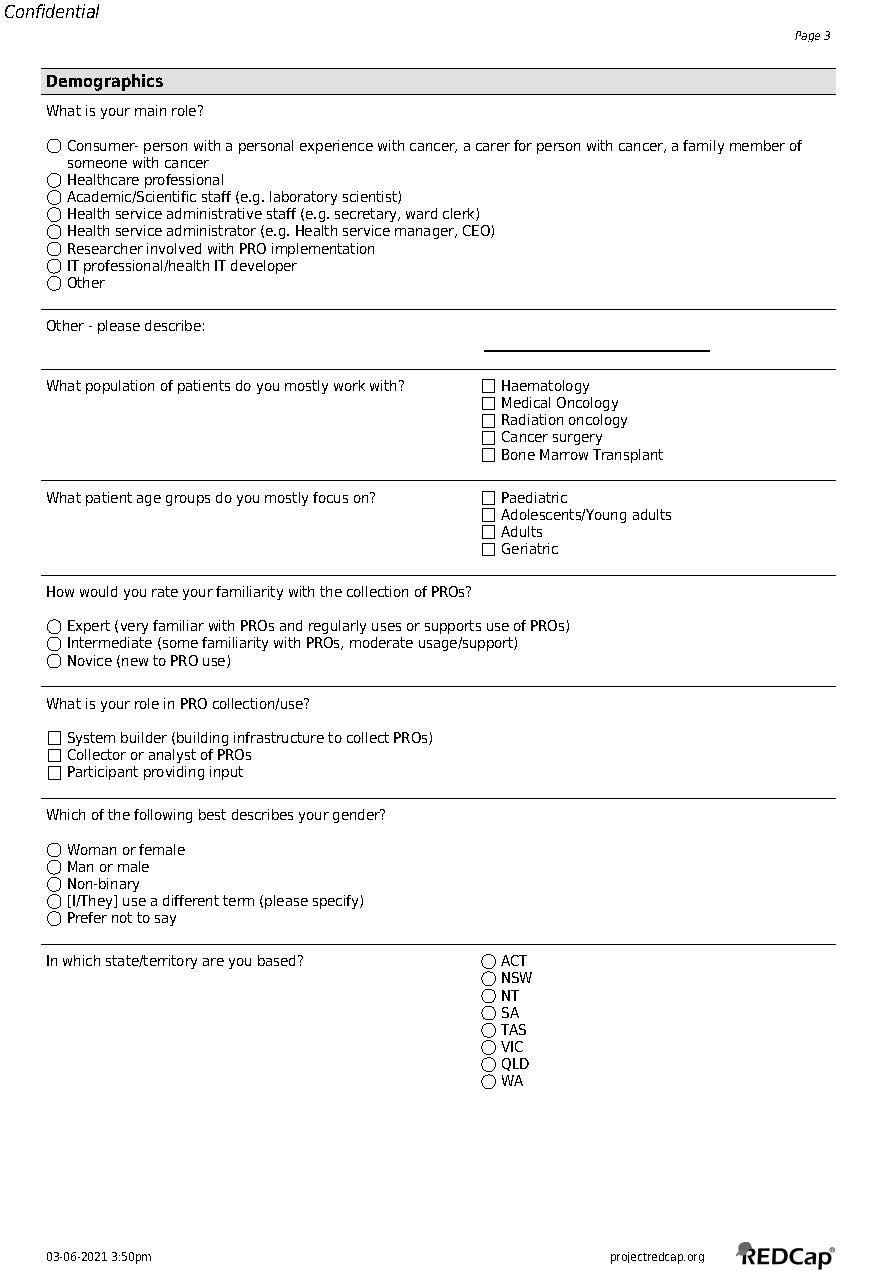

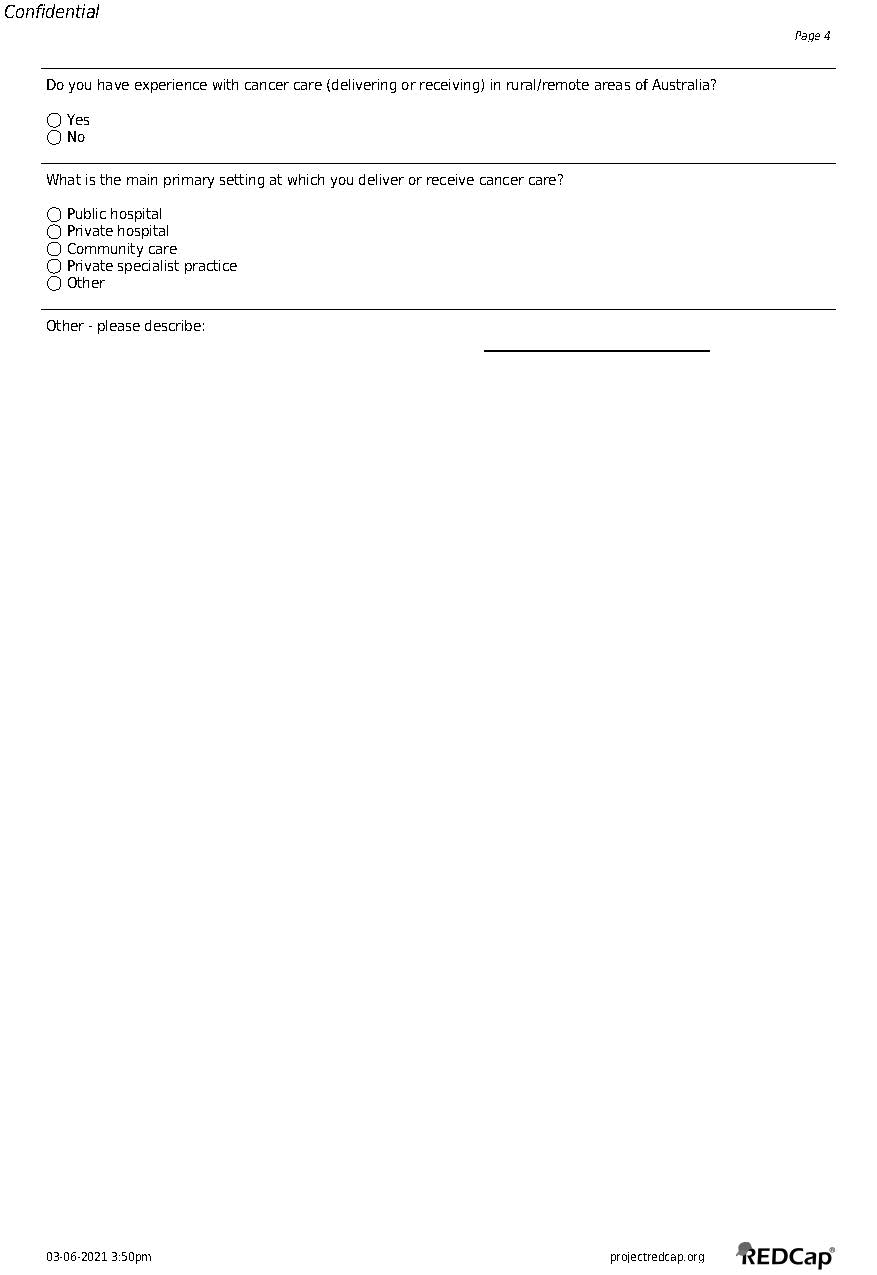

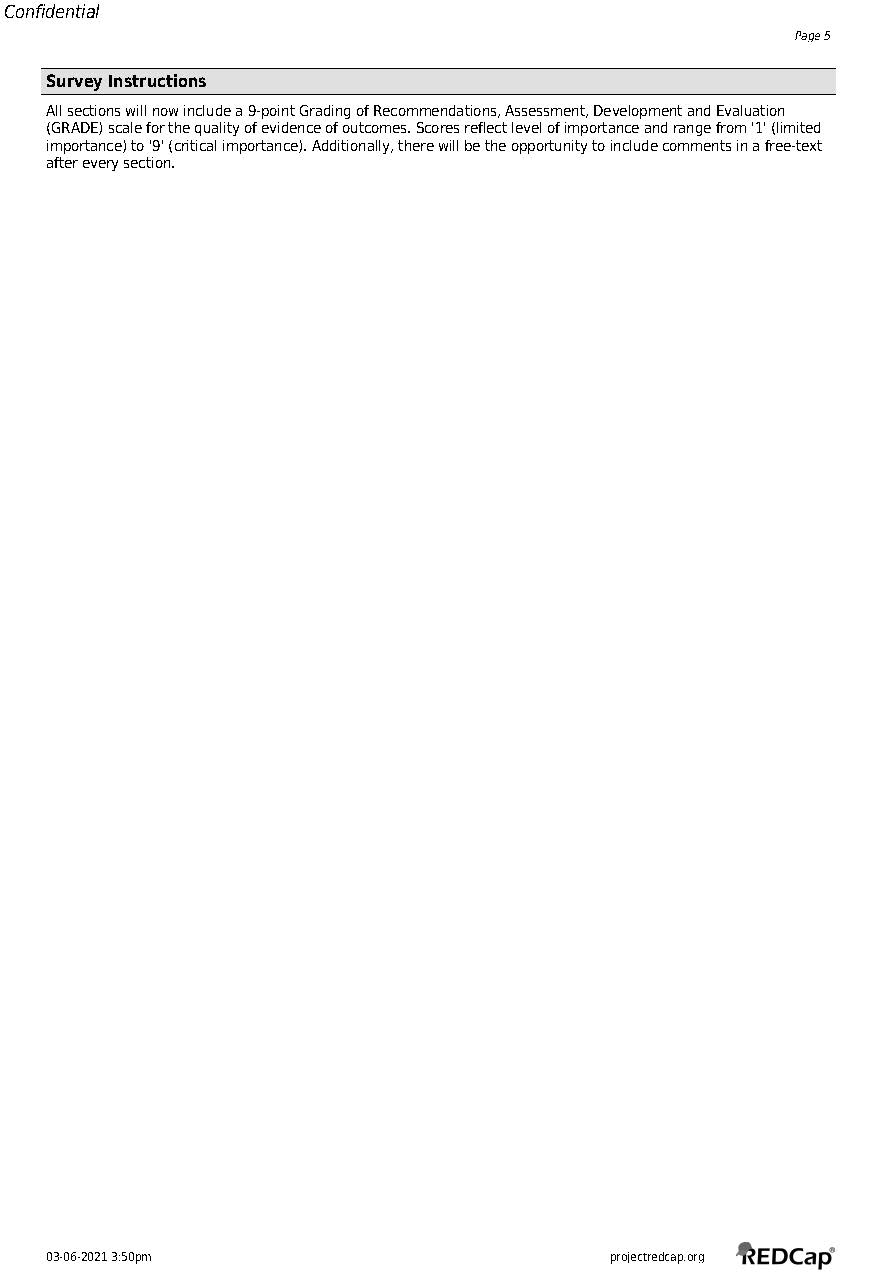

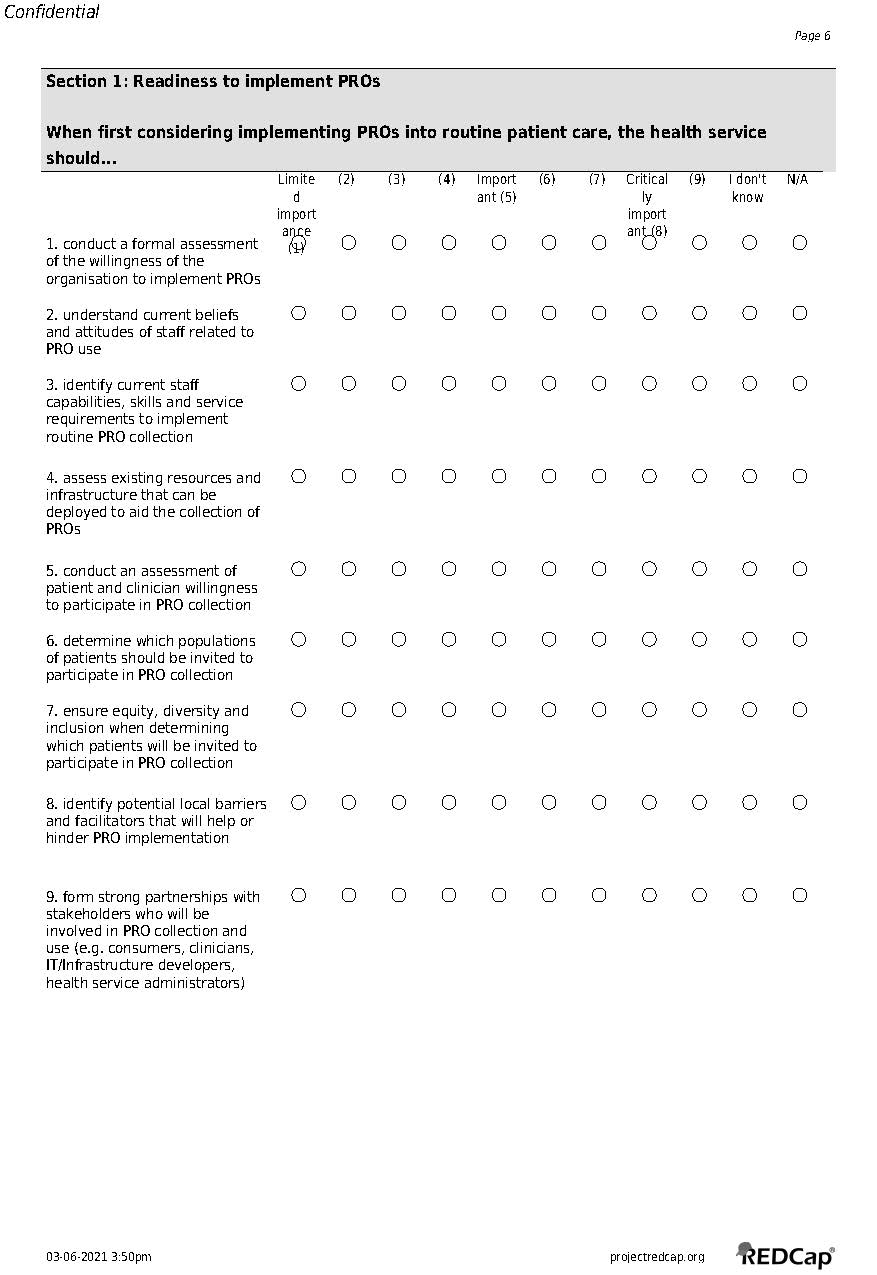

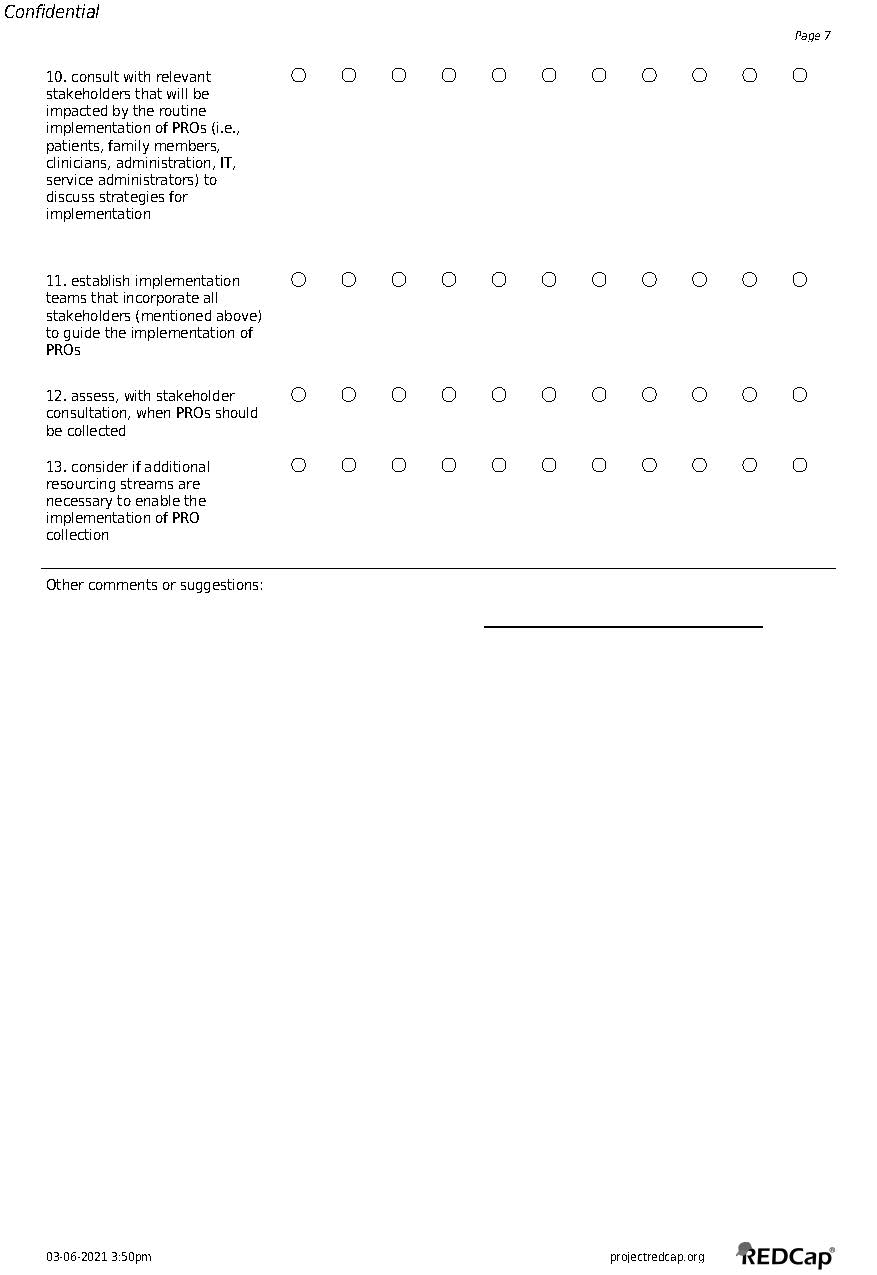

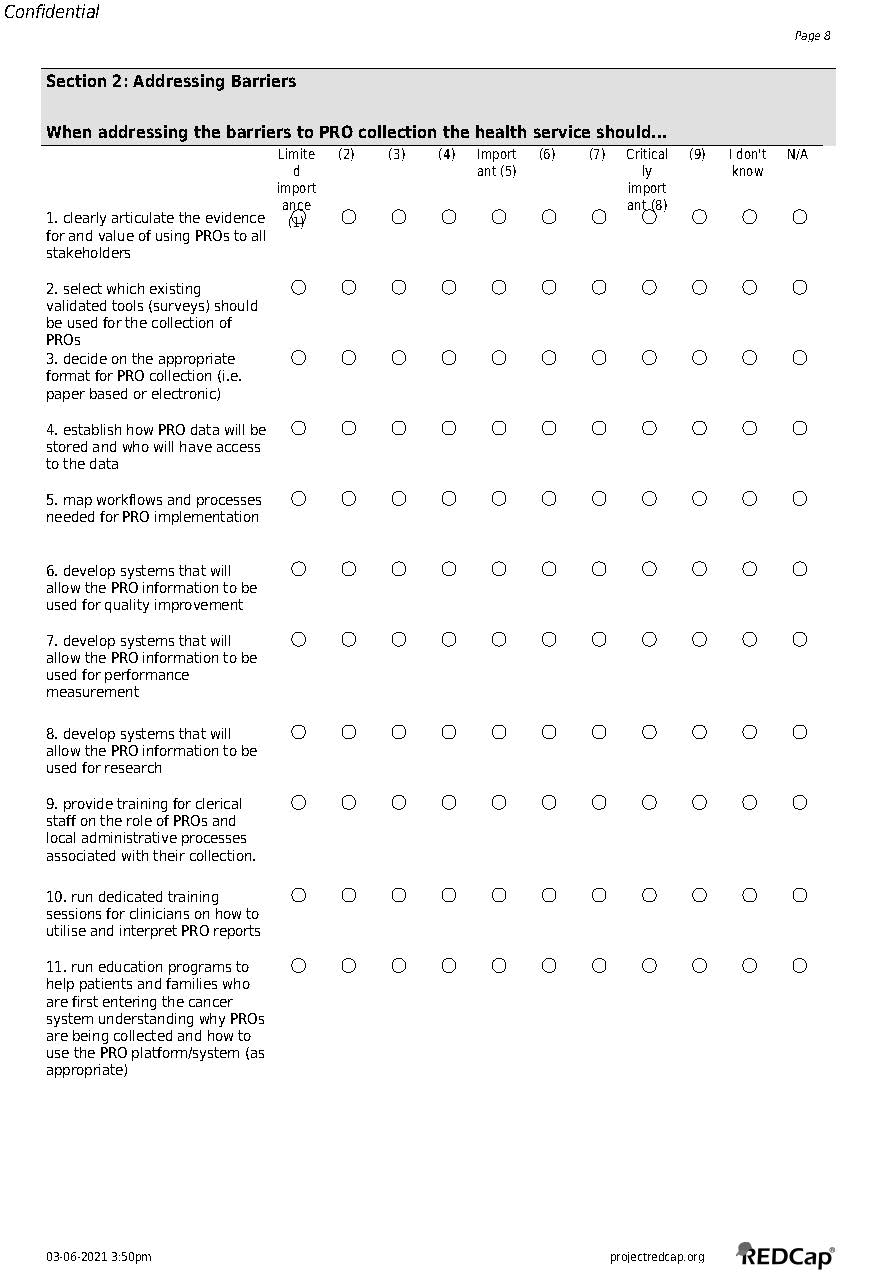

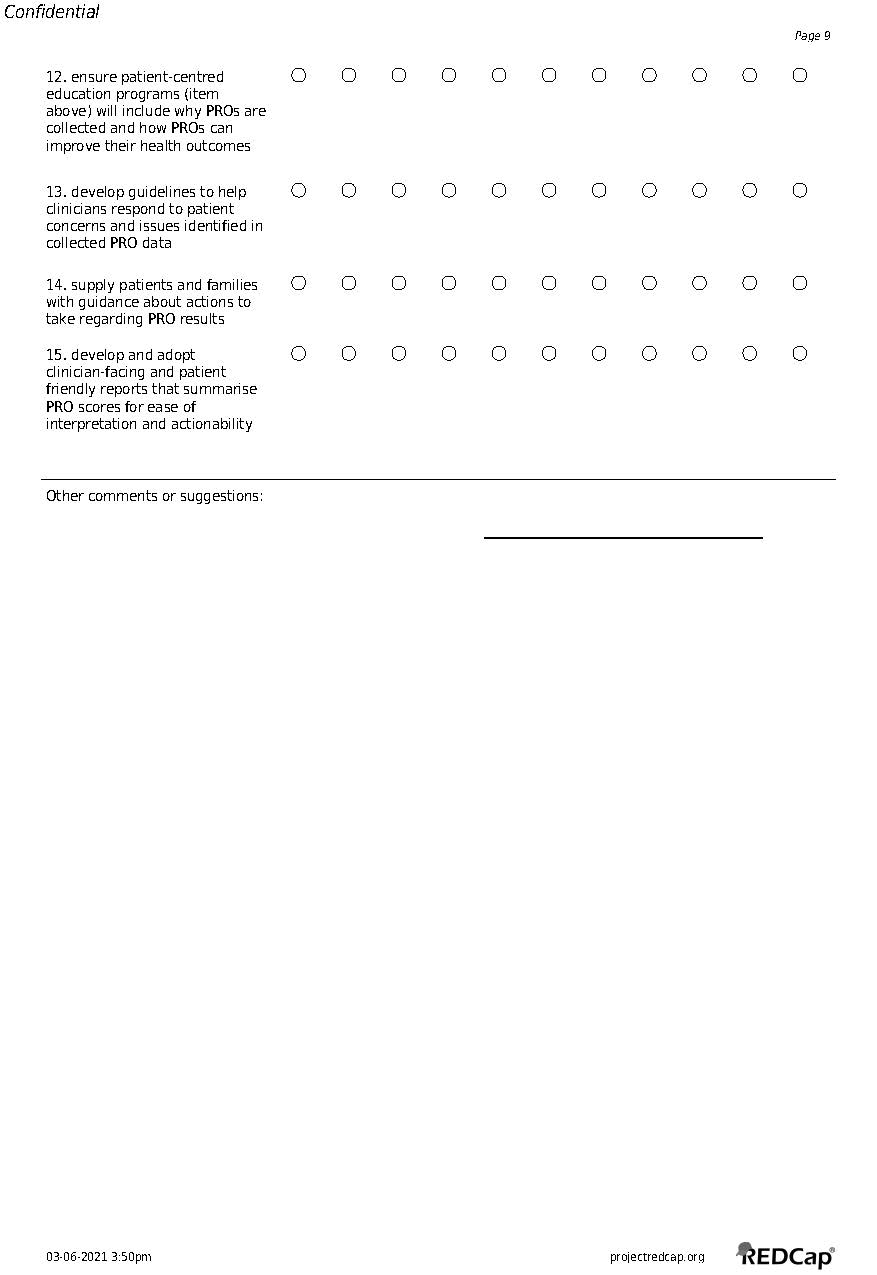

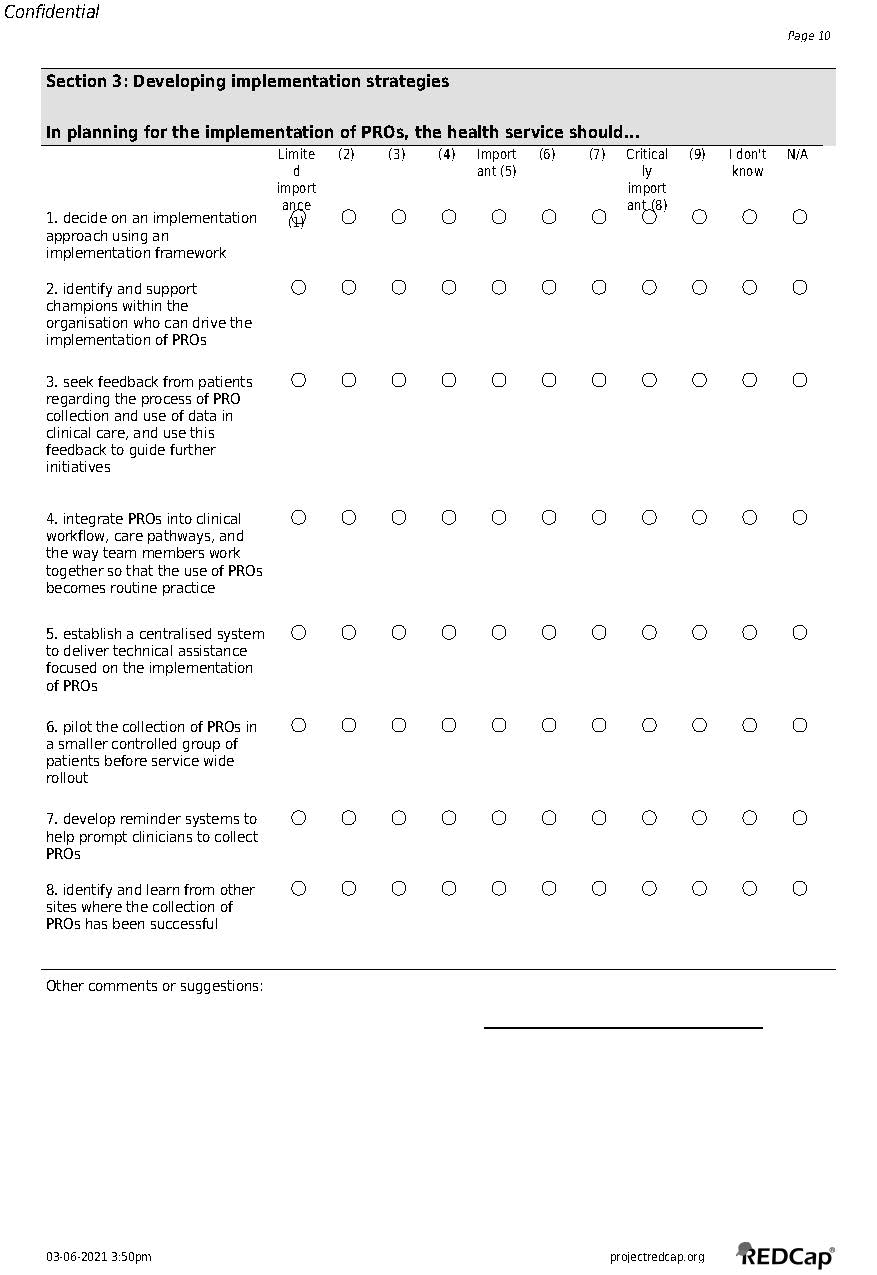

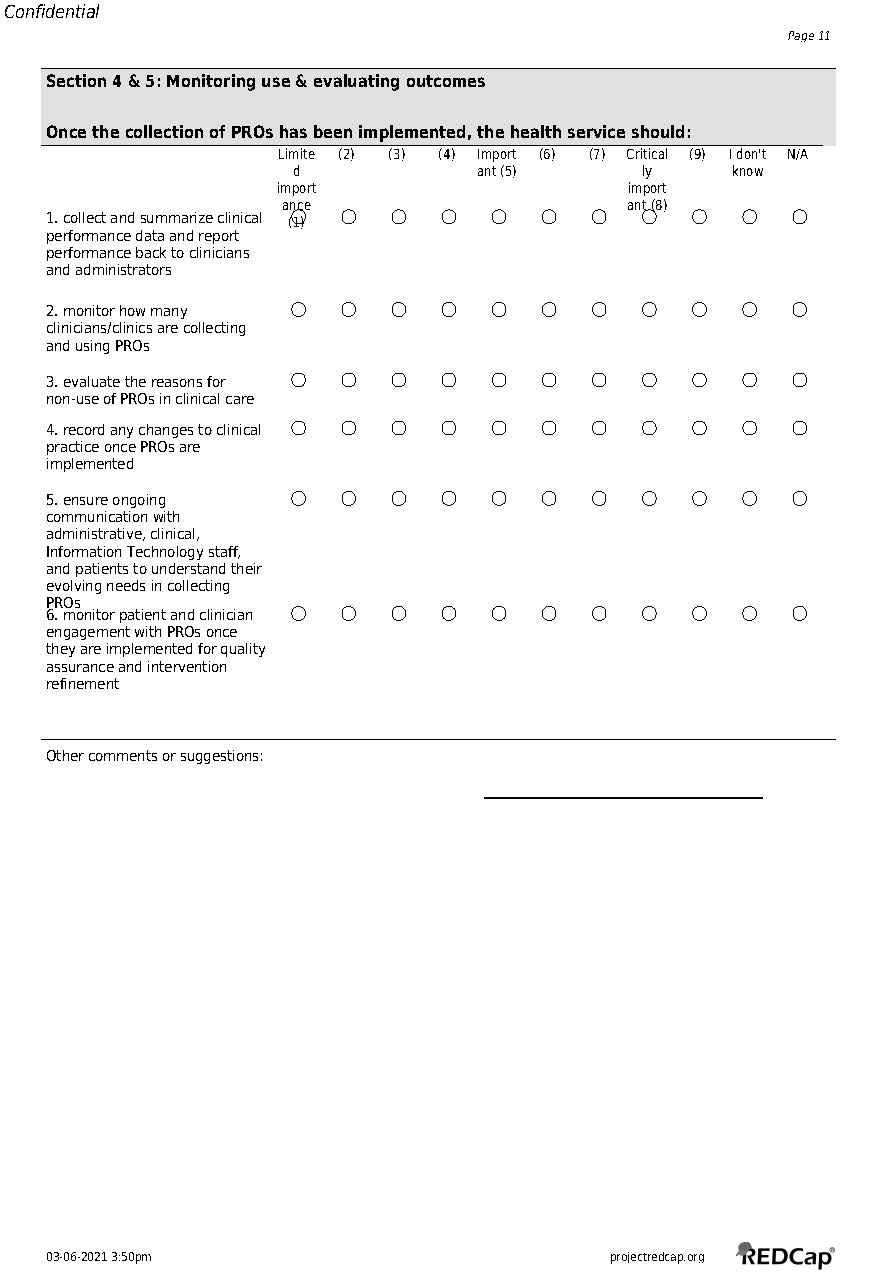

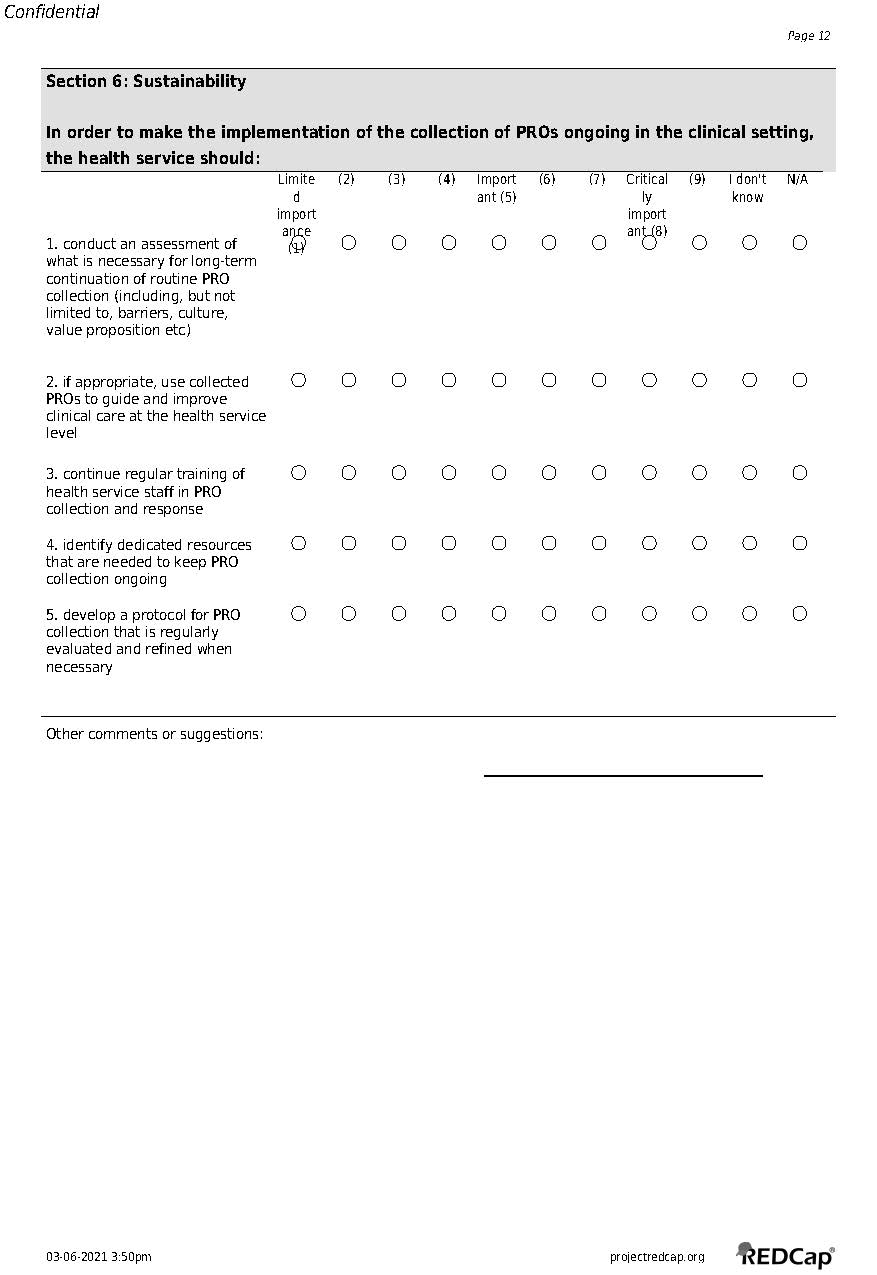


**Appendix E:** Round 2 survey
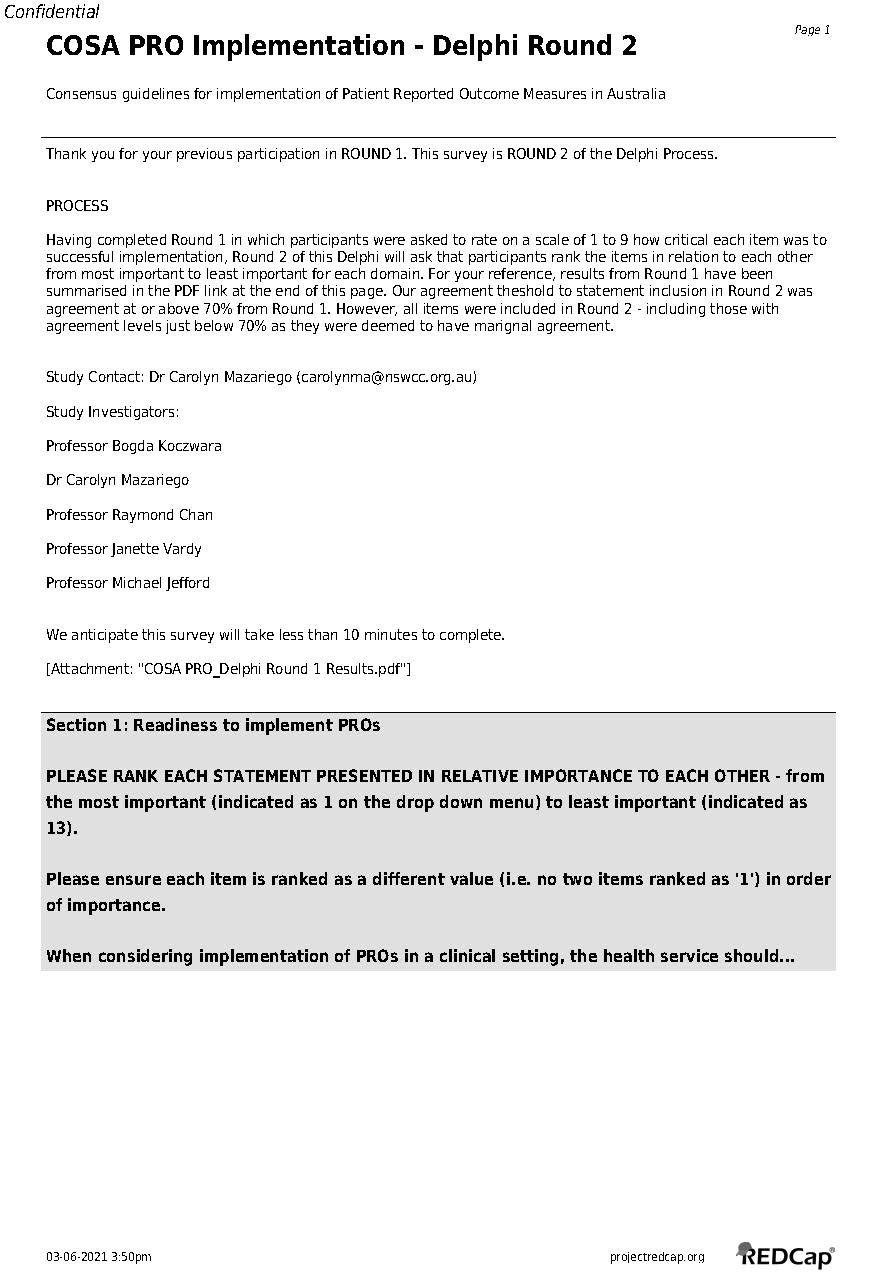

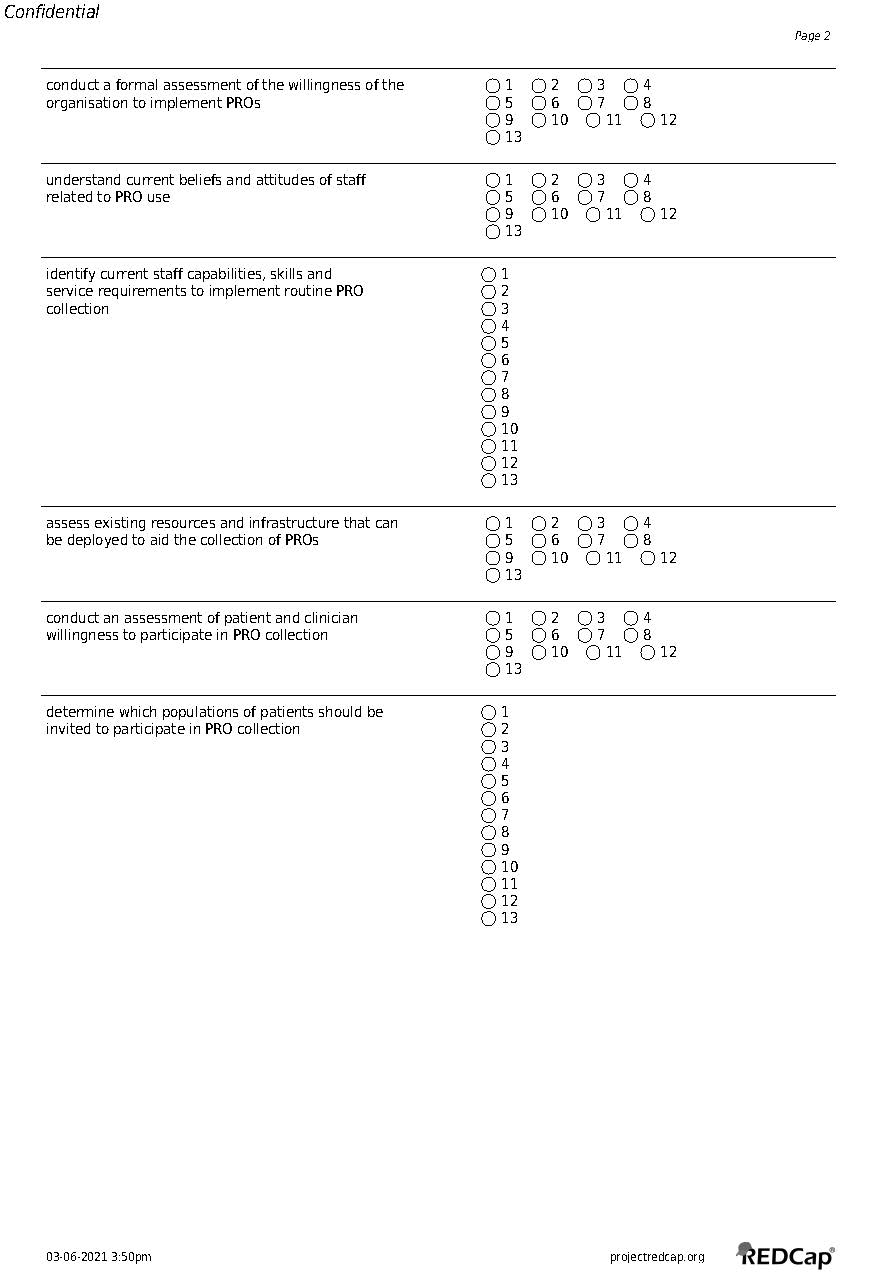

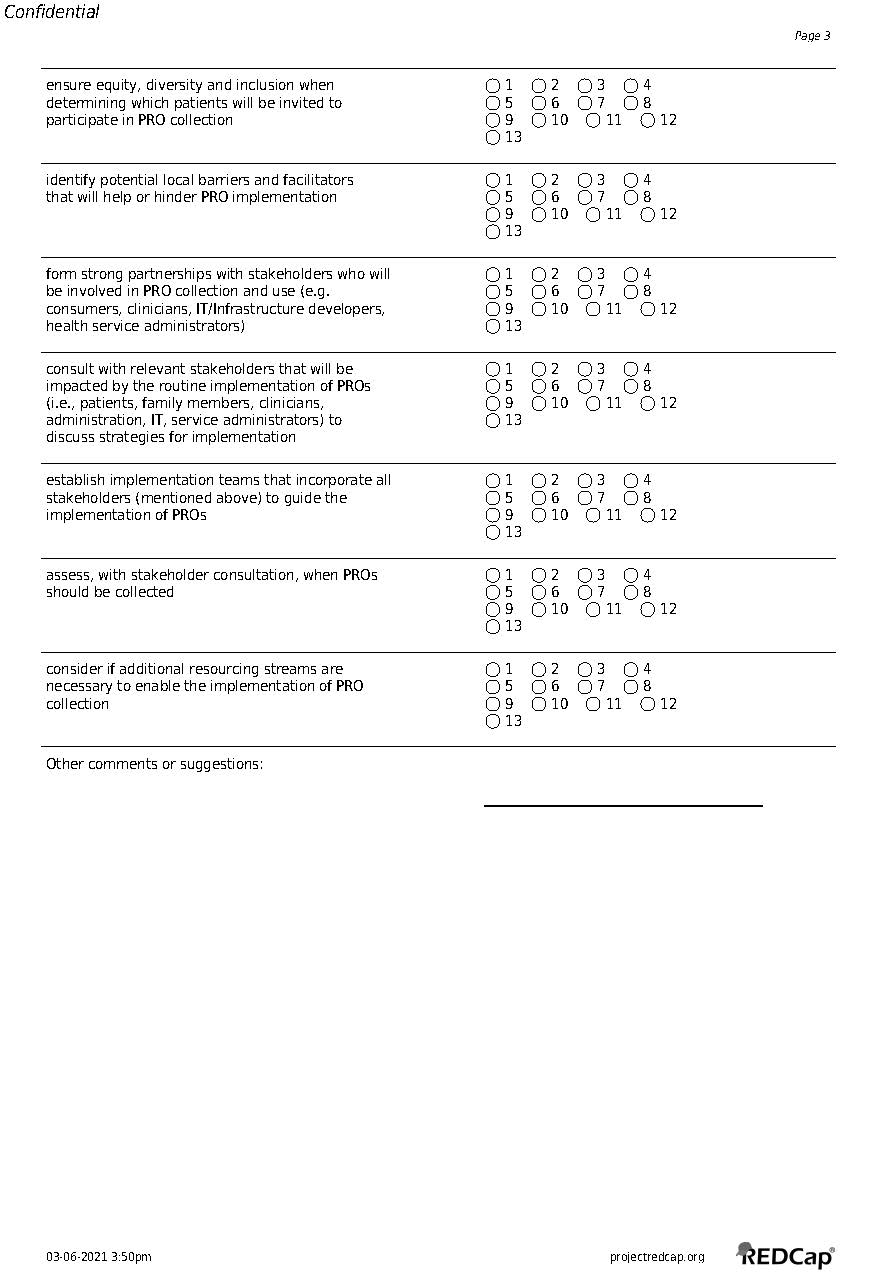

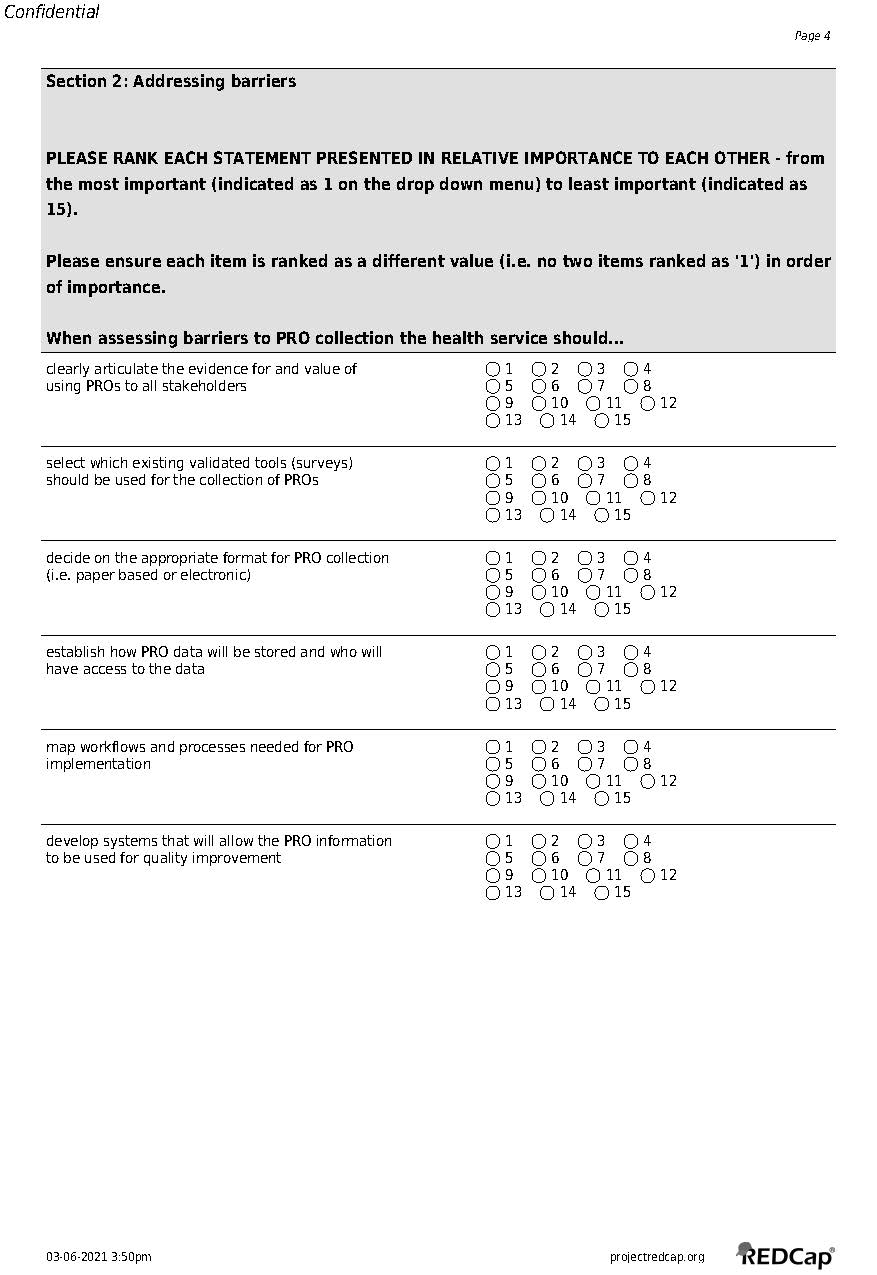

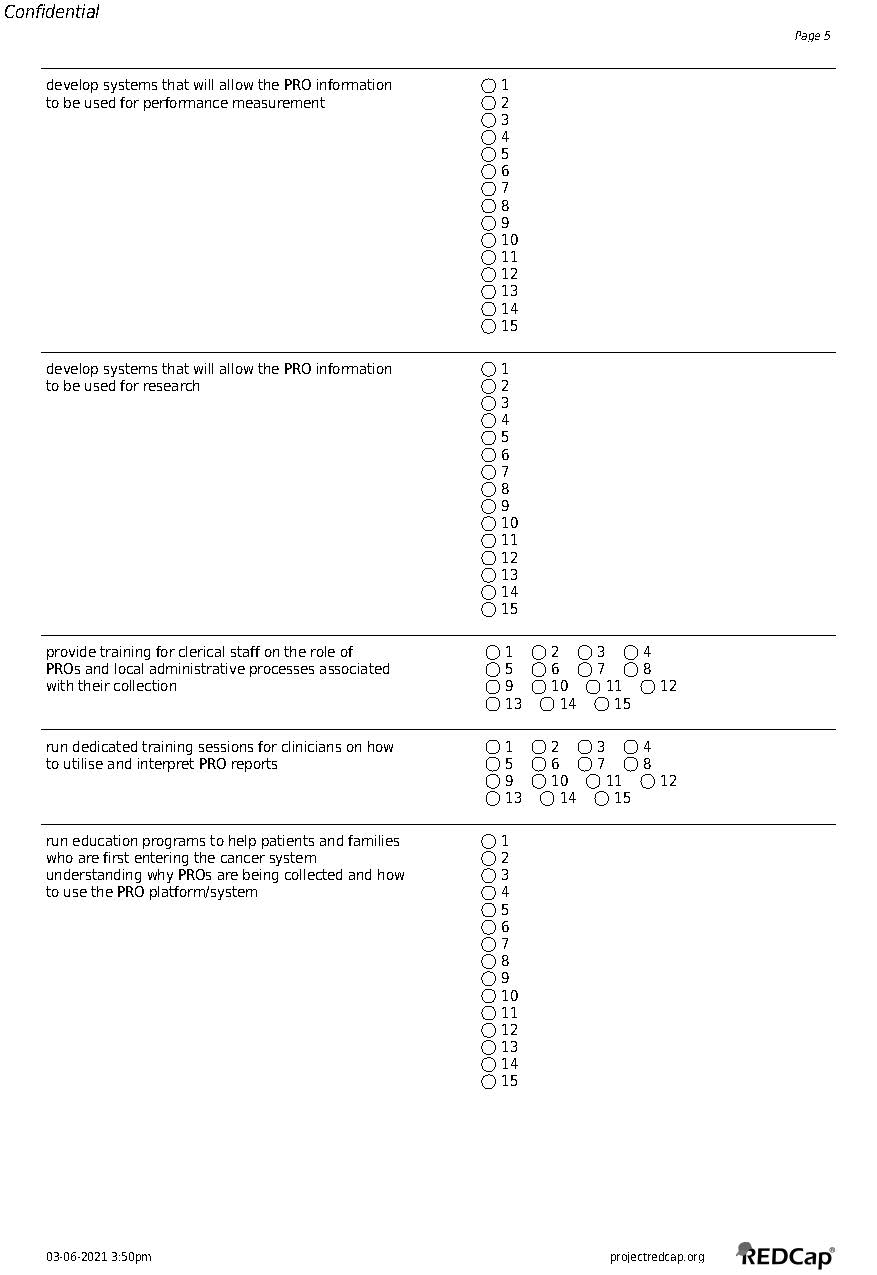

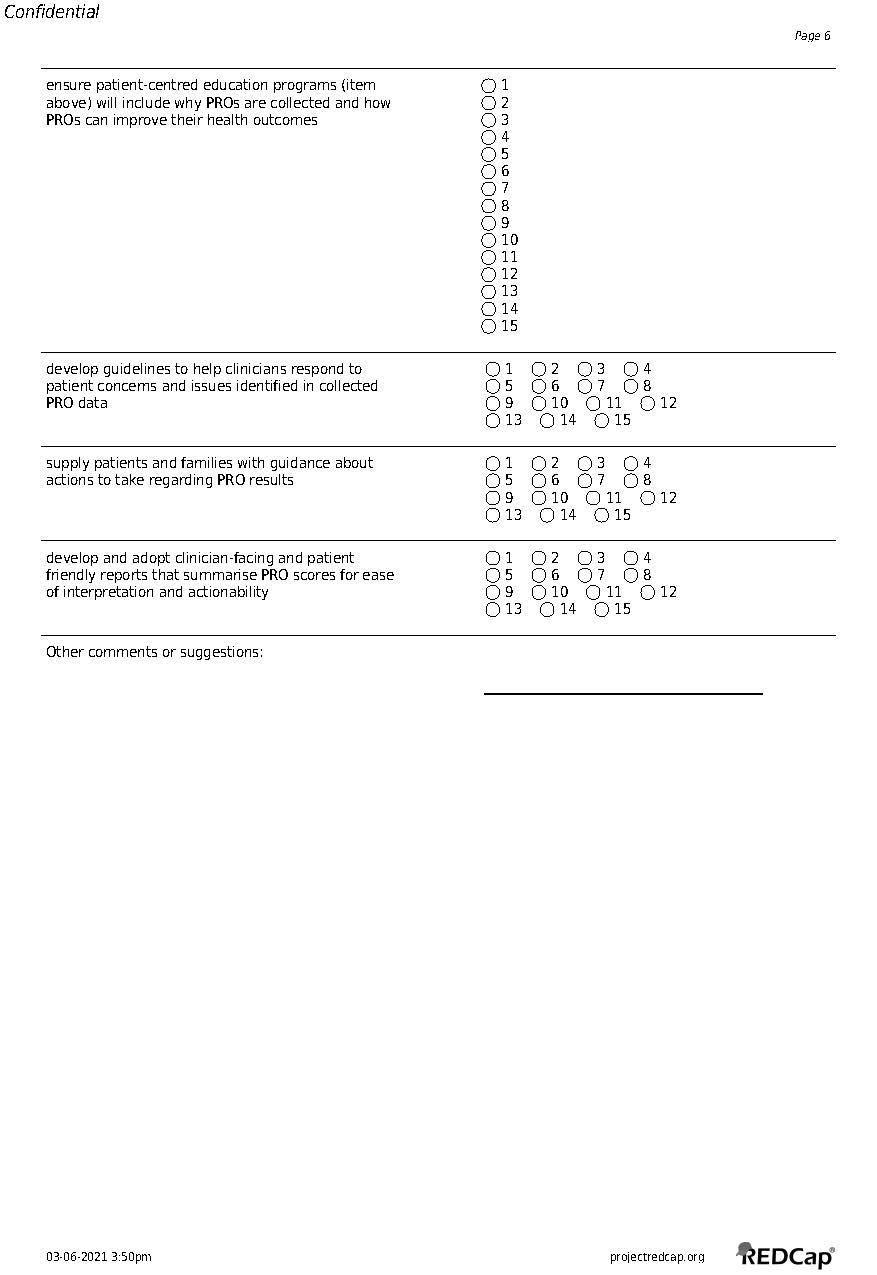

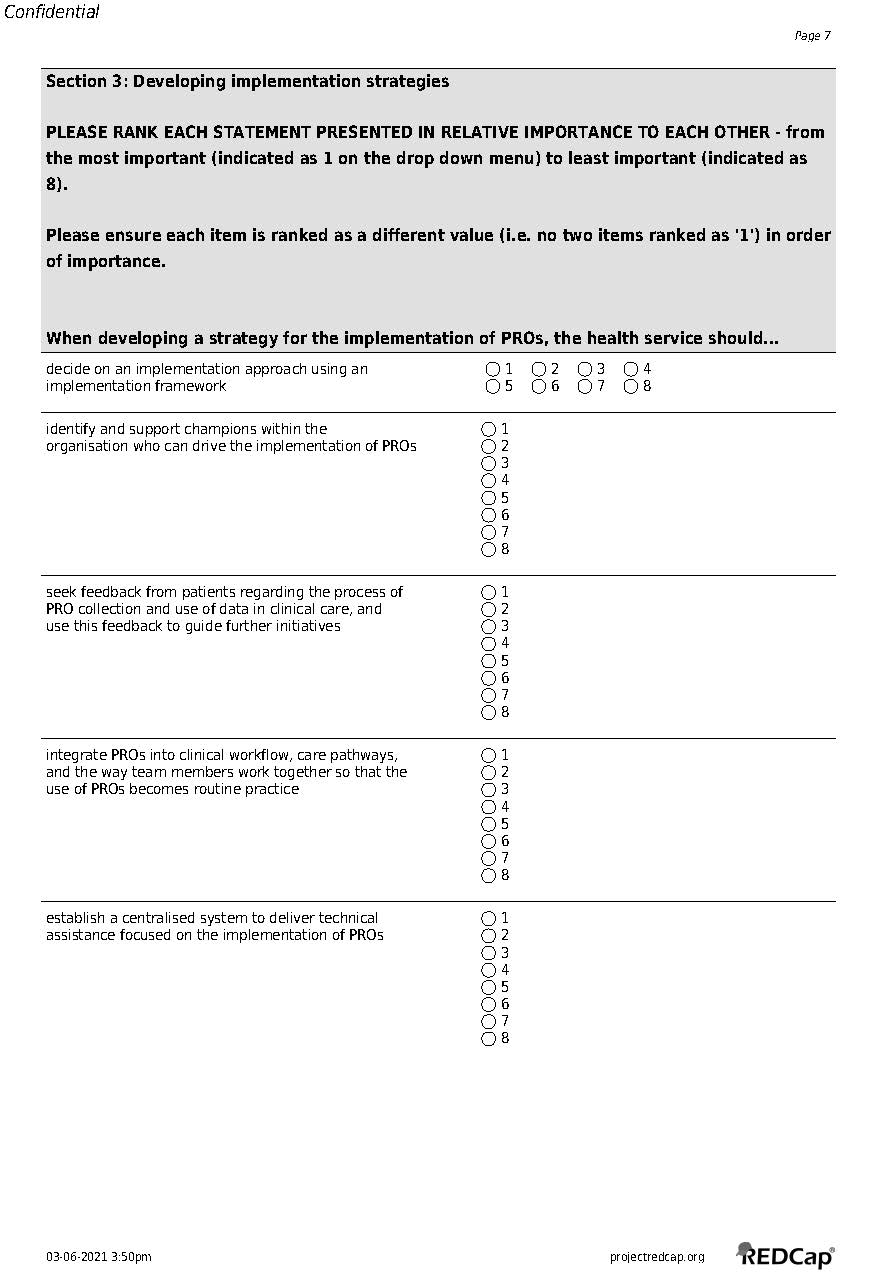

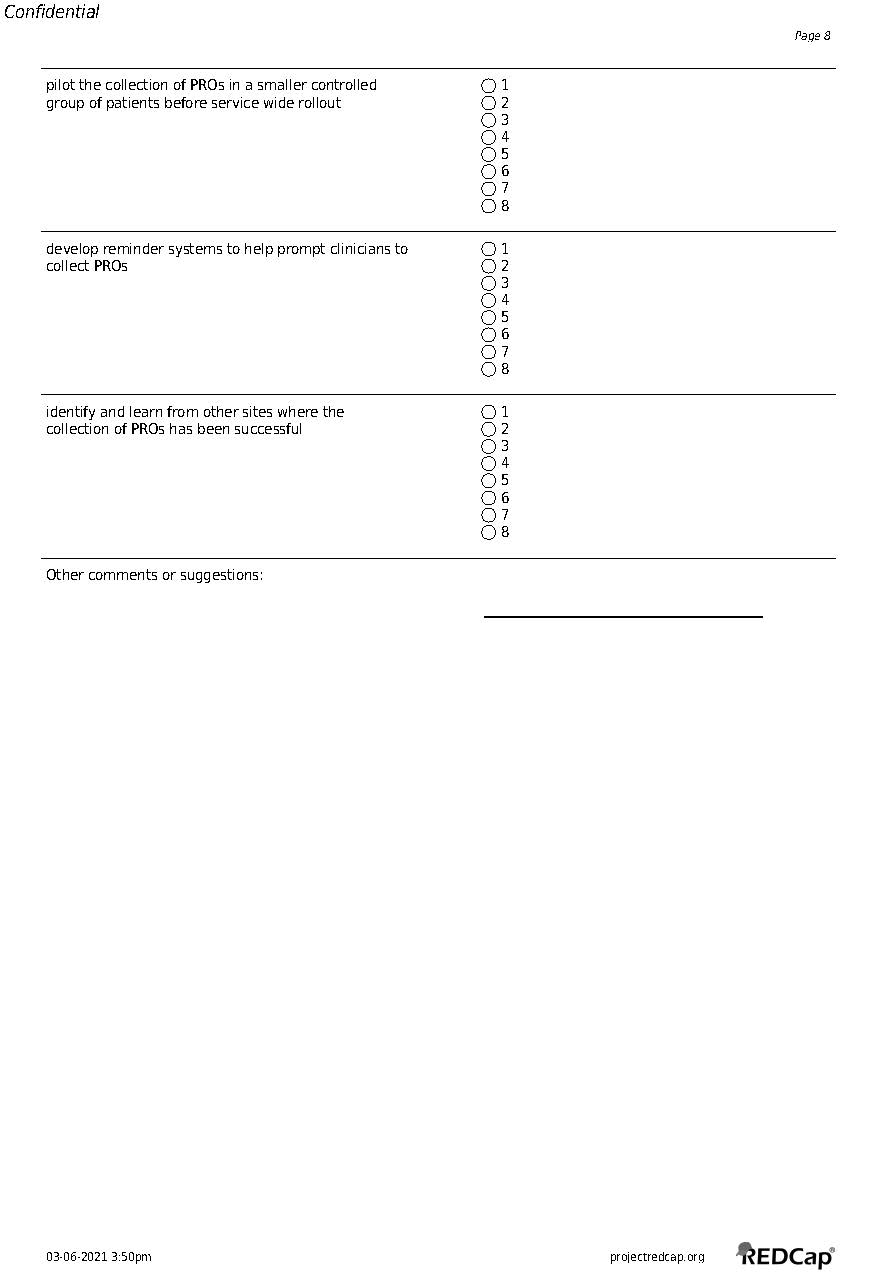

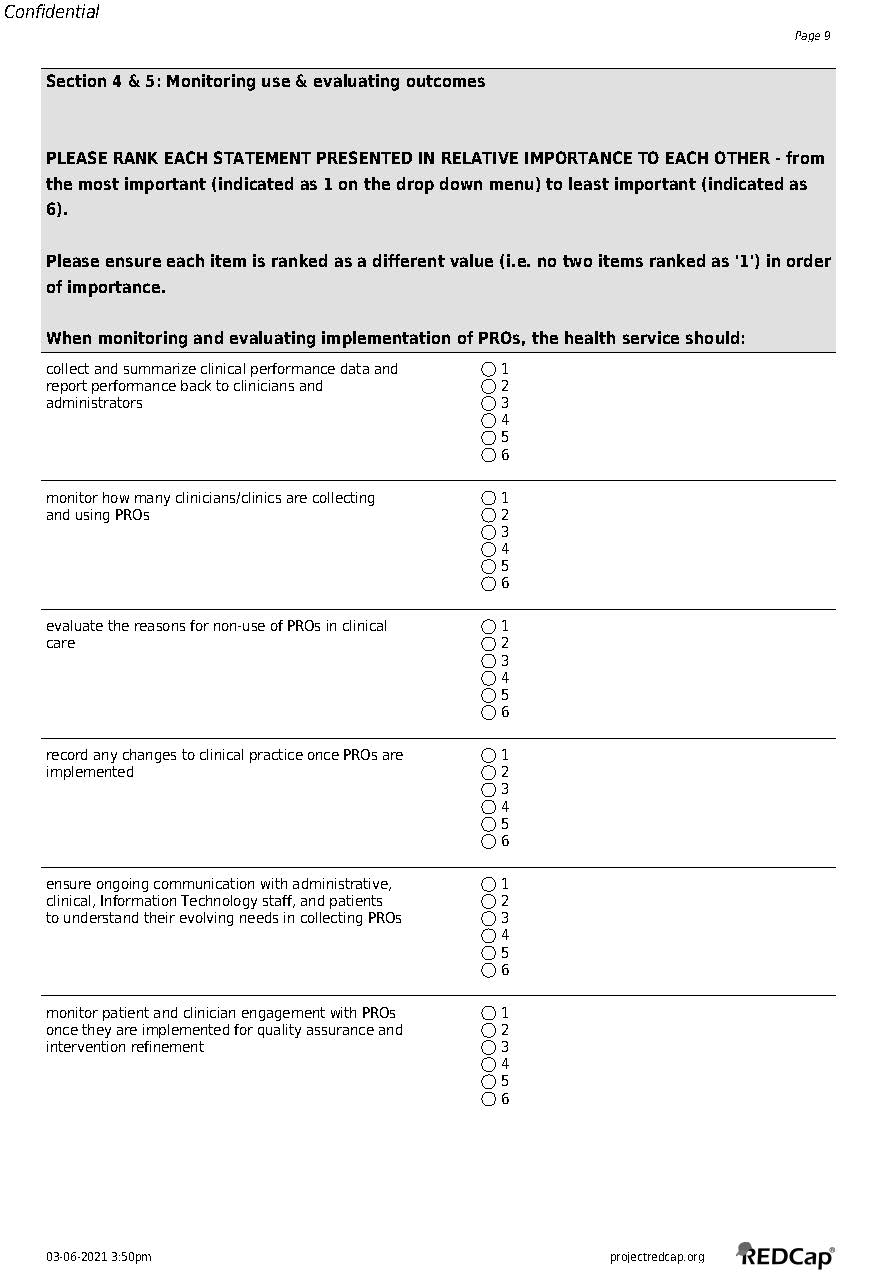

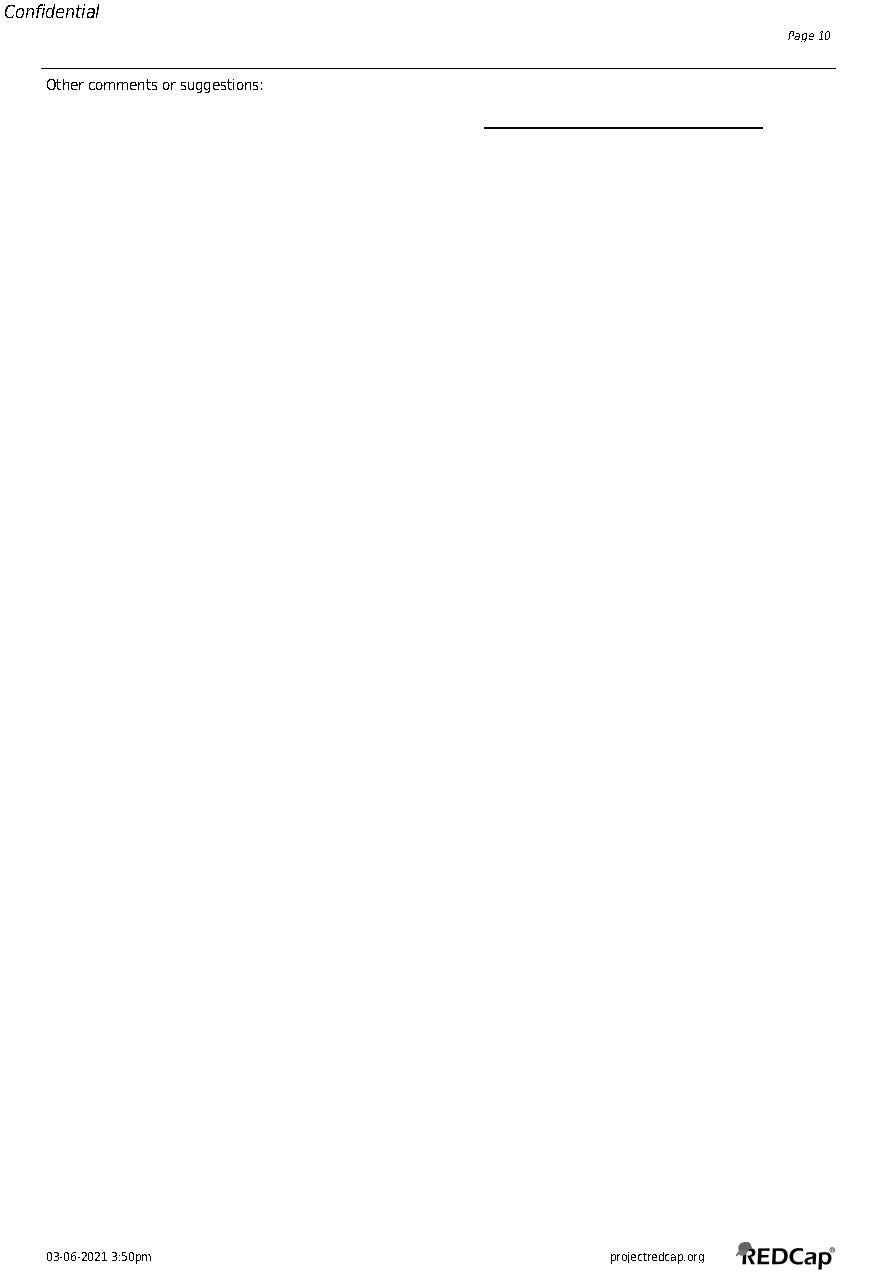

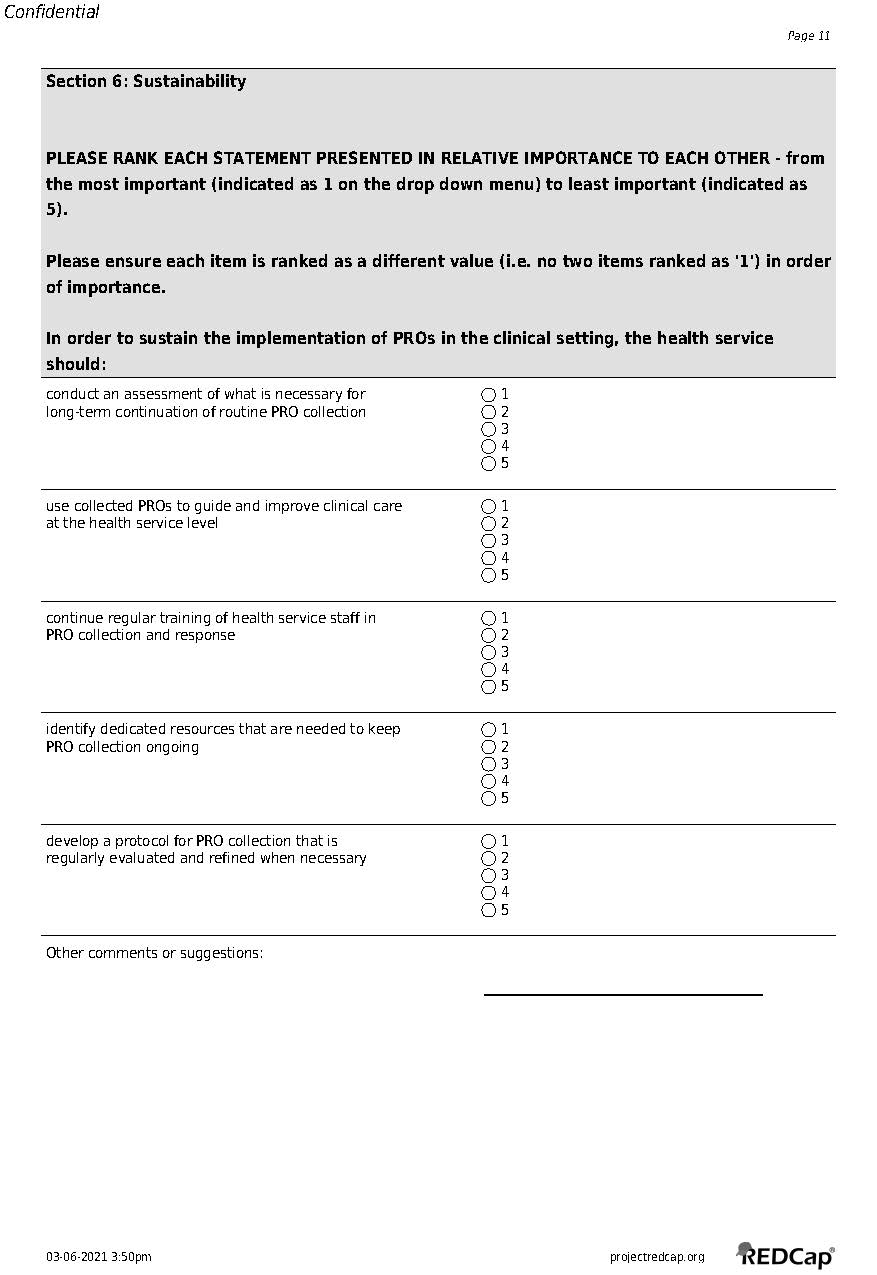


**Appendix F:** Draft priority statement generation list from literature review on PRO implementation

General statements

1. Dedicated resources (including human, financial, health systems) should be invested to integrate PROs into clinical care, given their demonstrated value and benefits
2. An Australian national PROs body consisting of PROs experts is needed to guide expert direction in all areas of healthcare, policy, and research.
3. The application of PROs must incorporate specific tools and strategies as needed to address equity, diversity, and inclusion.
4. PROs must be routinely collected and integrated to support continuous learning, improvement, and patient-centred decision-making in health care delivery.
5. conduct a formal assessment of the willingness and capability of the organisation to implement PROs.
6. understand current beliefs and attitudes related to PRO use.
7. identify current staff capabilities, skills and service requirements to implement routine PRO collection.
8. assess existing resources and infrastructure that can be deployed to aid the collection of PROs
9. conduct an assessment of patient and clinician willingness to participate in PRO collection.
10. determine which populations of patients should participate in PRO collection.
11. ensure equity, diversity and inclusion when determining which patients will participate in PRO collection.
12. identify potential local barriers and facilitators that will help or hinder PRO implementation
13. form strong partnerships with those implementing PRO collection (e.g. consumers, clinicians, IT/Infrastructure developers, health service administrators).
14. consult with relevant stakeholders that will be impacted by the routine implementation of PROs (i.e., patients, family members, clinicians, administration, IT, service administrators) to discuss strategies for implementation.
15. establish implementation teams that incorporate all stakeholders to guide the implementation of PROs
16. assess, with stakeholder consultation, when PROs should be collected.

PROs for Health Policy

1. Decision-making about access to care (including current and new technologies, drugs, or therapies) should be guided by a deliberative approach that incorporates PROs.
2. Both National, state and local bodies need to be established to guide selection of PRO measures and interpretation of PRO data for action and decision-making.
3. Routine collection and integration of PROs can be operationalized through a learning health system model in which every patient and every encounter provides an opportunity for PRO data collection to support improvement initiatives.
4. We must create a national repository of anonymized data provided by validated PROs tools for health care quality improvement
5. We must integrate the use of PROs into the core health care curricula
6. We must include the implementation and adoption of PROs as a mandatory requirement of a health care institution or facility accreditation

Selecting relevant PROs

1. We must engage with patients as partners from the start, together with family advisors and clinicians to select disease-specific PROMs that are meaningful for the patient, clinically relevant, and appropriate for clinical research.
2. A patient-oriented approach has all aspects of PROs evaluation, including selection, implementation, research and evaluation.
3. We must integrate PROs into clinical workflow, care pathways, and the way team members work together so that the use of PROs becomes routine practice
4. PROs must be captured before and between each clinical encounter to document adverse events and other patient issues, and configure workflow to ensure response to the data.
5. We must invest in electornic capture methods for PROs that integrate with patient records.
6. We must include documentation systems and analytics in PROs programs to enable data abstraction for quality improvement, performance measurement, and research.

Clinicians use of PROs in a real-world setting for person-centred care

1. We must educate clinicians initially and on a continuing basis on how to interpret PRO scores, to integrate those data into everyday patient management, and to understand the benefits of use.
2. Clinicians require specific and ongoing practice coaching for integrating PROs into shared decision-making and treatment planning.
3. We must create intuitive clinician-facing reports that summarise PRO scores for ease of interpretation and actionability.
4. We must align PRO scores and evidence in best-practice guidelines that direct clinicans to the selection and application of optimal clinical interventions to address detected problems.
5. We must include mechanisms for evaluating the implementation of PROs in patient management and for ongoing quality improvement.
6. There must be a clinical response each and every time PROs are collected.

Supporting patients to become informed partners in achieving better health outcomes

1. We must create an inclusive and accessible education program to help patients and families who are first entering the cancer system learn why pros are collected and how pros can improve their health outcomes.
2. Patients and families need to learn how to interpret pros scores and score changes, and they need guidance about actions to take and the use of the data for communicating with the health care team.
3. Patient and families need to learn that pros can be used for symptom monitoring, early intervention, informing treatment plans, understanding disease patterns and health recovery, and guiding self-management behaviours.
4. We must develop visually appealing patient-friendly pros reports for easy interpretation and an understanding about how pros changes align with disease and treatment changes.
5. We must integrate pros data with patient portals so that patients can link pros data with clinical and laboratory data, and complete pros between clinic visits.

PROs for Research

1. Agreement on a minimum common dataset should be established to permit comparative research and to allow for synergies with health policy and other research groups.
2. Computer-adaptive methods can facilitate collection of both core and disease-specific proms, and psychometric research about the clinical application of such an approach is needed.
3. Although research about the additional effect of pro measurement is required, reduced symptom burden might not be a clinically plausible outcome for evaluation.
4. PROs can be used for adverse event reporting, and proms designed to measure adverse effects of a therapy should be administered in the setting most suitable for the patient, at the time that the adverse effects are most prominent and top of the patient’s mind so that the patient perspective is captured more fully.
5. PROs data collected in the clinic should be reviewed before patient consultation and used to guide and improve clinical care.
6. Health and quality-of-life data have to be captured in addition to treatment-induced side effects that might require supportive care measures.
7. Clinical guidelines have to be in place to guide clinician responses to patient concerns and issues identified in the patient’s data during the clinic visit.
8. We should consider adding a section on the PROs questionnaire for completion by the family. Such a section could be helpful for both the patient and the care partner who is playing the vital role of caring for the patient. Completion of this part of the questionnaire might also identify whether supportive care services are required for the person supporting the patient.
9. Standardizing nationally-collected pros data might help to standardize clinical practice. Currently, there is no consistency from one cancer centre to another in the proms that are administered.

Communicate the value of PROs

1. clearly articulate the evidence for and value of using PROs to all stakeholders

Tools for PRO collection

1. select existing validated tools (surveys) to use for the collection of PROs
2. decide on the appropriate format for PRO collection (i.e. paper based or electronic)

Process

1. establish how PRO data will be stored and who will have access to the data
2. ensure that the interpretation of PRO data is incorporated into the clinical workflow
3. map workflows and processes
4. develop systems that will allow the PRO information to be used for quality improvement, performance measurement, and research

Training

1. provide training for clerical staff on the role of PROs and local administrative processes associated with their collection.
2. run dedicated training sessions for clinicians on how to utilise and interpret PRO reports
3. run education programs to help patients and families who are first entering the cancer system understanding why PROs are being collected and how to use the PRO platform/system (as appropriate)
4. ensure patient-centred education programs (item above) will include why PROs are collected and how PROs can improve their health outcomes

Response

1. collect and summarize clinical performance data and report performance back to clinicians and administrators
2. monitor how many clinicians/clinics are collecting and using PROs
3. evaluate the reasons for non-use (i.e.,patient preference or missed opportunity)
4. record any changes to clinical practice once PROs are implemented
5. ongoing communication with administrative, clinical, and Information Technology staff, as well as patients to understand evolving needs in collecting PROs
6. continuously monitor patient and clinician engagement with PROs once they are implemented for quality assurance and intervention refinement

1. develop or adopt guidelines to help guide clinician responses to patient concerns and issues identified in the patient’s PRO data
2. supply patients and families with guidance about actions to take regarding PRO results
3. develop and adopt clinician-facing and patient friendly reports that summarise PRO scores for ease of interpretation and actionability
4. develop or adopt guidelines to help guide clinician responses to patient concerns and issues identified in the patient’s PRO data
5. supply patients and families with guidance about actions to take regarding PRO results
6. develop and adopt clinician-facing and patient friendly reports that summarise PRO scores for ease of interpretation and actionability
